# Branched‐Chain Amino Acids Deficiency Promotes Diabetic Neuropathic Pain Through Upregulating LAT1 and Inhibiting Kv1.2 Channel

**DOI:** 10.1002/advs.202402086

**Published:** 2024-07-01

**Authors:** Ze‐Yu Zhou, Ji‐Ying Wang, Zhi‐Xiao Li, Hong‐Li Zheng, Ya‐Nan Zhou, Li‐Na Huang, Li‐Juan Wang, Xiao‐Wei Ding, Xin Sun, Ke Cai, Rui Zhao, Yan Shi, Alex F. Chen, Zhi‐Qiang Pan, Jing Cao, Fu‐Qing Lin, Jian‐Yuan Zhao

**Affiliations:** ^1^ State Key Laboratory of Genetic Engineering School of Life Sciences Fudan University Shanghai 200438 China; ^2^ Institute for Developmental and Regenerative Cardiovascular Medicine MOE‐Shanghai Key Laboratory of Children's Environmental Health Xinhua Hospital Shanghai Jiao Tong University School of Medicine Shanghai 200092 China; ^3^ Department of Pain Medicine Shanghai Tenth People's Hospital Tongji University School of Medicine Shanghai 200072 China; ^4^ Department of Human Anatomy School of Basic Medical Sciences Zhengzhou University Zhengzhou 450001 China; ^5^ Department of Anesthesiology Shanghai General Hospital Shanghai Jiao Tong University School of Medicine Shanghai 20080 China; ^6^ Jiangsu Province Key Laboratory of Anesthesiology Jiangsu Province Key Laboratory of Anesthesia and Analgesia Application Technology NMPA Key Laboratory for Research and Evaluation of Narcotic and Psychotropic Drugs Xuzhou Medical University Xuzhou 221004 China

**Keywords:** Kv1.2, branched‐chain amino acids, diabetic neuropathic pain, L‐type amino acid transporter 1

## Abstract

Diabetic neuropathic pain (DNP), one of the most common complications of diabetes, is characterized by bilateral symmetrical distal limb pain and substantial morbidity. To compare the differences  is aimed at serum metabolite levels between 81 DNP and 73 T2DM patients without neuropathy and found that the levels of branched‐chain amino acids (BCAA) are significantly lower in DNP patients than in T2DM patients. In high‐fat diet/low‐dose streptozotocin (HFD/STZ)‐induced T2DM and leptin receptor‐deficient diabetic (db/db) mouse models, it is verified that BCAA deficiency aggravated, whereas BCAA supplementation alleviated DNP symptoms. Mechanistically, using a combination of RNA sequencing of mouse dorsal root ganglion (DRG) tissues and label‐free quantitative proteomic analysis of cultured cells, it is found that BCAA deficiency activated the expression of L‐type amino acid transporter 1 (LAT1) through ATF4, which is reversed by BCAA supplementation. Abnormally upregulated LAT1 reduced Kv1.2 localization to the cell membrane, and inhibited Kv1.2 channels, thereby increasing neuronal excitability and causing neuropathy. Furthermore, intraperitoneal injection of the LAT1 inhibitor, BCH, alleviated DNP symptoms in mice, confirming that BCAA‐deficiency‐induced LAT1 activation contributes to the onset of DNP. These findings provide fresh insights into the metabolic differences between DNP and T2DM, and the development of approaches for the management of DNP.

## Graphical abstract

Treatment of diabetic neuropathic pain remains a major challenge due to its unclear mechanisms. This paper describes an observation that branched‐chain amino acids (BCAA) abundance is correlated with diabetic neuropathic pain in patients, and offers new perspectives on the metabolic regulation of diabetic neuropathic pain beyond traditional glucose‐centric approaches.

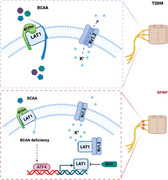

## 1 Introduction

Type 2 diabetes mellitus (T2DM) is the most common, complex, and comprehensive metabolic disease that causes varying degrees of damage to different tissues of the body.^[^ ^1^, ^2^ ^]^ Diabetic polyneuropathy is the most common complication of diabetes and ≈50% of patients with diabetes eventually suffer from neuropathy.^[^ ^3^ ^]^ In a 25‐year cohort study, the prevalence of clinically diagnosed diabetic neuropathy was ≈45%, but increased to 60–75% when patients were diagnosed using more sensitive nerve conduction assays.^[^ ^3^, ^4^ ^]^

Diabetic neuropathic pain (DNP), characterized by bilateral symmetrical distal limb pain, is a common symptom of neuropathy. At disease onset, DNP is more common distally in the feet and gradually develops in the proximal leg and hand, with severe pain at night.^[^ ^3^ ^]^ The pathogenesis and underlying mechanisms of DNP have not been fully elucidated, and there are no clinical diagnostic markers. Current approaches to the management of DNP rely on glycemic control (mainly in patients with T1DM), diet and lifestyle interventions (mainly in patients with T2DM), nutritional therapy for nerves, and commonly used analgesics.^[^ ^3^, ^5^, ^6^ ^]^ However, many clinical studies have shown that there is no strong evidence that glycemic control can significantly improve DNP, especially in patients with T2DM. Glycemic control is ineffective in reducing pain symptoms,^[^ ^3^, ^4^, ^5^, ^7^, ^8^, ^9^ ^]^ suggesting that factors other than glycemic metabolites contribute to DNP development. Therefore, understanding the metabolic differences between DNP and T2DM without neuropathy is fundamental to reveal the pathophysiological factors underlying DNP and develop new therapeutic targets.

Branched‐chain amino acids (BCAA), including leucine, isoleucine, and valine, are essential for the human body.^[^ ^10^ ^]^ BCAA supplementation or a BCAA‐rich diet has important positive effects on body weight, muscle protein synthesis, and glucose homeostasis.^[^ ^10^, ^11^ ^]^ Because the levels of circulating BCAA tend to be elevated in obese individuals and are associated with the deterioration of metabolic health and future insulin resistance in T2DM,^[^ ^11^, ^12^ ^]^ BCAA have long been recognized as a risk factor for T2DM. Nevertheless, rats fed a low‐fat diet supplemented with BCAA do not develop insulin resistance.^[^ ^11^ ^]^ A BCAA dysmetabolism model proposes that the accumulation of downstream metabolites (not BCAA per se) promotes β‐cell mitochondrial dysfunction.^[^ ^12^ ^]^

The idea that BCAA supplementation directly contributes to the development of metabolic diseases remains controversial, and BCAA itself may not be sufficient to cause the disease. Conversely, BCAA intraventricular injection can alleviate a series of behavioral abnormalities in mice such as decreased desire to explore, disordered gait, and reduced social activities.^[^ ^13^ ^]^ A recent study indicated that BCAA are essential for perinatal neuronal metabolic state and survival.^[^ ^14^ ^]^ These results suggest a putative positive effect of BCAA on the nervous system.

The cellular uptake of BCAA mainly facilitated by the L‐type amino acid transporter (LAT1), also known as SLC7A5, which forms a heterodimer with the 4F2 cell‐surface antigen heavy chain (4F2hc), also known as SLC3A2.^[^ ^15^, ^16^, ^17^, ^18^, ^19^, ^20^ ^]^ In the heterodimer, 4F2hc appears to stabilize the scaffolding domain of LAT1 in the membrane, and LAT1 is responsible for the recognition and uptake of substrates.^[^ ^19^ ^]^ Loss of LAT1 in the endothelial cells of blood vessels and the blood‐brain barrier results in a significant decrease in BCAA levels in the brains of adult mice, while other large neutral amino acid levels are comparable to those observed in the control. These findings suggest that LAT1 is critical for the maintenance of normal brain BCAA levels.^[^ ^13^ ^]^ This study also showed that LAT1 loss of function leads to autism spectrum disorders and motor dysfunctions in humans and mice and that intracerebroventricular administration of BCAA ameliorates abnormal behaviors in mice.^[^ ^13^ ^]^ Furthermore, the deletion of LAT1 in neural progenitor cells affects the postnatal metabolic state, neuronal survival, and persistent behavioral defects.^[^ ^14^ ^]^

Herein, in our retrospective observational case‐control study, we showed that human plasma samples from DNP patients, compared to T2DM, displayed reduced BCAA levels. In high‐fat diet/low‐dose streptozotocin (HFD/STZ)‐induced T2DM and leptin receptor‐deficient diabetic (db/db) mouse models, we found that BCAA deficiency in the mouse dorsal root ganglion (DRG) led to compensatory upregulation of LAT1 levels. Pathological dysregulation of LAT1 inhibits Kv1.2 channels, thereby increasing neuronal excitability and contributing to the occurrence of DNP.

## 2 Results

### 2.1 Decreased Plasma BCAA Levels Are Associated with DNP

To investigate whether the metabolite profiles of patients with T2DM are associated with the risk of DNP, 194 patients were screened. Of these, 81 patients with DNP and 73 age‐ and sex‐matched patients with T2DM but without neuropathy fulfilled the inclusion criteria. Thirty age‐ and sex‐matched healthy individuals were recruited as healthy controls. The clinical characteristics of all individuals are presented in Table S1 (Supporting Information). There were no differences in the duration of diabetes, body mass index, fasting blood glucose (FBG) levels, or other indications between patients with T2DM and DNP. This suggests that metabolites other than blood glucose are also involved in DNP development. There were no differences in medication use between the groups (Table S2, Supporting Information).

Fasting plasma samples were collected and metabolite profiling was performed using nuclear magnetic resonance (NMR) as we previously described.^[^ ^21^, ^22^, ^23^ ^]^ Of the successfully identified and quantified metabolites, the concentrations of BCAA (leucine, isoleucine, and valine) were most significantly associated with DNP. The levels of isoleucine, leucine, and valine in the DNP patients were decreased by 15.09% (*p* = 7.44E‐7), 13.37% (*p* = 4.78E‐7), and 12.93% (*p* = 8.18E‐7), respectively, compared to the levels in the T2DM patients (**Table** **1**). Among these, isoleucine showed the most significant change.

**Table 1:** Plasma metabolite concentration in different groups was measured by NMR.

Metabolites [µmol L^−1^]	① Healthy People [*n* = 30]	② T2DM Patients [*n* = 73]	③ DNP Patients [*n* = 81]	② versus ① adjusted *p* value	③ versus ② adjusted *p* value
Isoleucine	130.62 ± 2.60	163.39 ± 3.85	138.73 ± 3.13	8.31E‐7	7.44E‐7
Leucine	136.39 ± 3.22	152.26 ± 3.00	131.91 ± 2.50	0.005	4.78E‐7
Valine	288.88 ± 5.85	321.86 ± 6.26	280.23 ± 5.36	0.005	8.18E‐7
Tyrosine	67.24 ± 2.25	64.29 ± 1.58	57.29 ± 1.31	0.794	0.003
Histidine	76.27 ± 1.61	65.53 ± 1.64	60.50 ± 1.34	3.11E‐4	0.040
Tryptophan	65.64 ± 2.46	53.23 ± 1.22	49.63 ± 1.08	7.76E‐7	0.115
Glutamine	755.48 ± 11.76	619.00 ± 15.42	590.25 ± 13.18	7.61E‐7	0.393
Phenylalanine	82.45 ± 1.64	74.28 ± 2.05	71.96 ± 1.50	0.033	0.986
Lactate	3296.64 ± 119.56	4057.14 ± 315.86	3508.57 ± 160.60	0.231	0.259
Alanine	634.15 ± 17.91	506.18 ± 15.50	499.97 ± 13.62	1.03E‐5	>0.999
Citrate	85.88 ± 1.70	117.11 ± 3.60	106.12 ± 2.74	2.23E‐7	0.026
Acetate	96.20 ± 1.50	103.31 ± 3.45	124.48 ± 4.88	>0.999	6.60E‐4
Creatine	86.05 ± 1.80	97.21 ± 2.14	91.43 ± 1.97	0.008	0.108
Formate	386.13 ± 6.70	217.65 ± 8.21	206.38 ± 10.26	<E‐15	>0.999
Hypoxanthine	13.54 ± 0.53	39.83 ± 7.18	28.44 ± 4.26	0.029	0.388
Inosine	4.89 ± 0.35	11.97 ± 2.14	14.21 ± 2.70	0.305	>0.999
3‐Hydroxybutytrate	226.66 ± 10.12	227.33 ± 12.75	220.44 ± 19.90	>0.999	>0.999

Values are expressed as mean ± SEM. One‐way analysis of variance (ANOVA) was used to analyze the differences among the three groups, followed by Bonferroni's multiple comparison test to obtain adjusted *p* values.

These results from human clinical samples indicate that decreased BCAA levels are associated with the onset of DNP. Although high BCAA levels are a risk factor for T2DM development, we found that BCAA exhibited a protective effect in patients with DNP who had already developed T2DM.

### 2.2 BCAA Deficiency Aggravates DNP Symptoms in Mice

**Figure 1:**
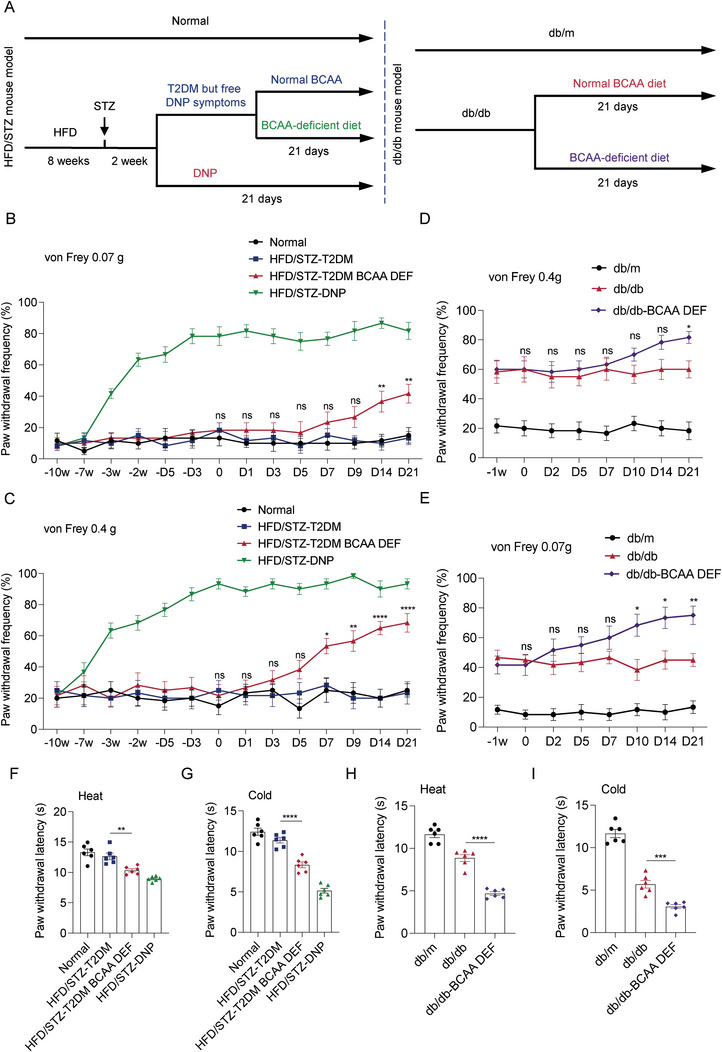
BCAA deficiency aggravates DNP symptoms in mice. A) Schematic representation of the establishment of two T2DM mouse models and BCAA deficiency feeding. B,C) The paw withdrawal frequencies (PWF) to 0.07 g (B) and 0.4 g (C) von Frey filaments were detected on the different days during the HFD/STZ mouse model establishment and BCAA deficiency feeding (*n* = 6 mice per group). D,E) The paw withdrawal frequencies (PWF) to 0.07 g (D) and 0.4 g (E) von Frey filaments were detected on different days during the BCAA deficiency feeding in db/db mice (*n* = 6 mice per group). F,G) The paw withdrawal latencies to heat (F) and cold (G) stimuli were detected after the BCAA deficiency feeding in HFD/STZ mice (*n* = 6 mice per group). H,I) The paw withdrawal latencies to heat (H) and cold (I) stimuli were detected after the BCAA deficiency feeding in db/db mice (*n* = 6 mice per group). Data are expressed as mean ± SEM. One‐way analysis of variance (ANOVA) was used to analyze the differences among three or more groups, followed by Bonferroni's multiple comparison test to obtain adjusted *p* values. Differences between the HFD/STZ‐T2DM and HFD/STZ‐T2DM BCAA DEF groups, and between the db/db and db/db‐BCAA DEF groups are shown. Significance is indicated as ^ns^ *p* > 0.05, ^*^ *p* < 0.05, ^**^ *p* < 0.01, ^***^ *p* < 0.001, and ^****^ *p* < 0.0001.

We first investigated whether decreased BCAA levels indeed increase the risk of DNP and are not only associated with the onset of DNP in high‐fat diet/low‐dose streptozotocin (HFD/STZ)‐induced T2DM and leptin receptor‐deficient diabetic (db/db) mouse models. The processes used to generate T2DM models and feeding programs are shown in **Figure** **1**A. The HFD/STZ mice were divided into two categories based on the presence or absence of DNP symptoms (mechanical allodynia). The subset of mice with T2DM symptoms that remained free of DNP symptoms were randomly divided into two groups, one group was given normal feeding (HFD/STZ‐T2DM group), and the other group was fed a BCAA‐deficient diet (HFD/STZ‐T2DM BCAA DEF group). The DNP symptomatic mice were given normal feeding and named the HFD/STZ‐DNP group. All db/db mice were randomly divided into two groups: one group was fed a normal diet (db/db group) and the other was fed a BCAA‐deficient diet (db/db‐BCAA DEF group). The beginning of feeding the BCAA deficiency diet was recorded as day 0, and the mice were tested for pain behavior on day 21.

All db/db mice showed mechanical allodynia, similar to those reported previously.^[^ ^24^, ^25^ ^]^ Blood and DRG samples from HFD/STZ mice were collected to detect BCAA levels, and it was found that BCAA levels were decreased in DNP mice compared with T2DM mice (Figure S1A,B, Supporting Information). This is consistent with the metabolomics results of clinical blood samples. Pain behavior tests showed that BCAA deficiency aggravated mechanical allodynia, demonstrated by increased paw withdrawal frequencies in response to low (0.07 g) and medium (0.4 g) force von Frey filaments (Figure 1B–E); heat hyperalgesia, and cold allodynia, evidenced by decreased paw withdrawal latencies in response to heat and cold stimuli, in HFD/STZ and db/db mouse models (Figure 1F–I). Twenty‐one days of BCAA deficiency did not alter body weight or fasting blood glucose levels in two mouse models (Figure S1C–F, Supporting Information). Taken together, these findings indicate that BCAA deficiency aggravates the symptoms of DNP in mice.

### 2.3 BCAA Supplementation Improves DNP Symptoms in Mice

**Figure 2:**
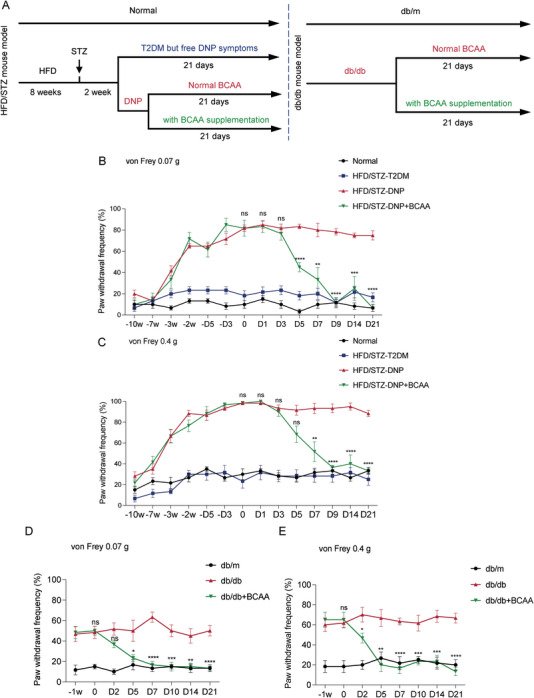
BCAA supplementation alleviates mechanical allodynia in HFD/STZ mice and db/db mice. A) Schematic representation of the establishment of two T2DM mouse models and BCAA supplementation. B,C) The paw withdrawal frequencies (PWF) to 0.07 g (B) and 0.4 g (C) von Frey filaments were detected on the different days during the HFD/STZ mouse model establishment and BCAA supplementation (*n* = 6 mice per group). D,E) The paw withdrawal frequencies (PWF) to 0.07 g (D) and 0.4 g (E) von Frey filaments were detected on different days during the BCAA supplementation in db/db mice (*n* = 6 mice per group). Data are expressed as mean ± SEM. One‐way analysis of variance (ANOVA) was used to analyze the differences among three or more groups, followed by Bonferroni's multiple comparison test to obtain adjusted *p* values. Differences between the HFD/STZ‐DNP and HFD/STZ‐DNP+BCAA groups, and between the db/db and db/db+BCAA groups are shown. Significance is indicated as ^ns^ *p* > 0.05, ^*^ *p* < 0.05, ^**^ *p* < 0.01, ^***^ *p* < 0.001, and ^****^ *p* < 0.0001.

Next, we explored whether BCAA supplementation alleviated DNP symptoms in HFD/STZ and db/db mouse models. The processes used to generate T2DM models and feeding programs are shown in **Figure** **2**A. The grouping method of the HFD/STZ mouse model was similar to that described above. The DNP symptomatic mice were randomly divided into two groups, one group was given normal feeding (HFD/STZ‐DNP group), and the other group was given BCAA supplementation (HFD/STZ‐DNP+BCAA group). All db/db mice were randomly divided into two groups: one group was fed a normal diet (db/db group) and the other was fed with BCAA supplementation (db/db+BCAA group). The beginning of BCAA supplementation was recorded as day 0, and the mice were tested for mechanical pain behavior at different times.

BCAA supplementation significantly attenuated mechanical allodynia in both mouse models, as demonstrated by decreased paw withdrawal frequencies in response to low (0.07 g) and medium (0.4 g) force von Frey filaments (Figure 2B–E). The db/db mice responded earlier and faster to BCAA supplementation than HFD/STZ‐DNP mice.

Twenty‐one days of BCAA supplementation did not alter the body weight in either mouse model compared to normal chow‐fed mice (Figure S2A,B, Supporting Information). In both mouse models, we examined the T2DM phenotypes, including hyperglycemia (Figure S2C,D, Supporting Information) and insulin resistance, as detected by glucose tolerance (Figure S2E,F, Supporting Information) and insulin tolerance (Figure S2G,H, Supporting Information) tests. Twenty‐one days of BCAA supplementation did not worsen T2DM symptoms in mice. Taken together, BCAA supplementation was beneficial in alleviating DNP symptoms in mice.

### 2.4 LAT1 Expression Levels Are Increased in the DRG of DNP Mice

**Figure 3:**
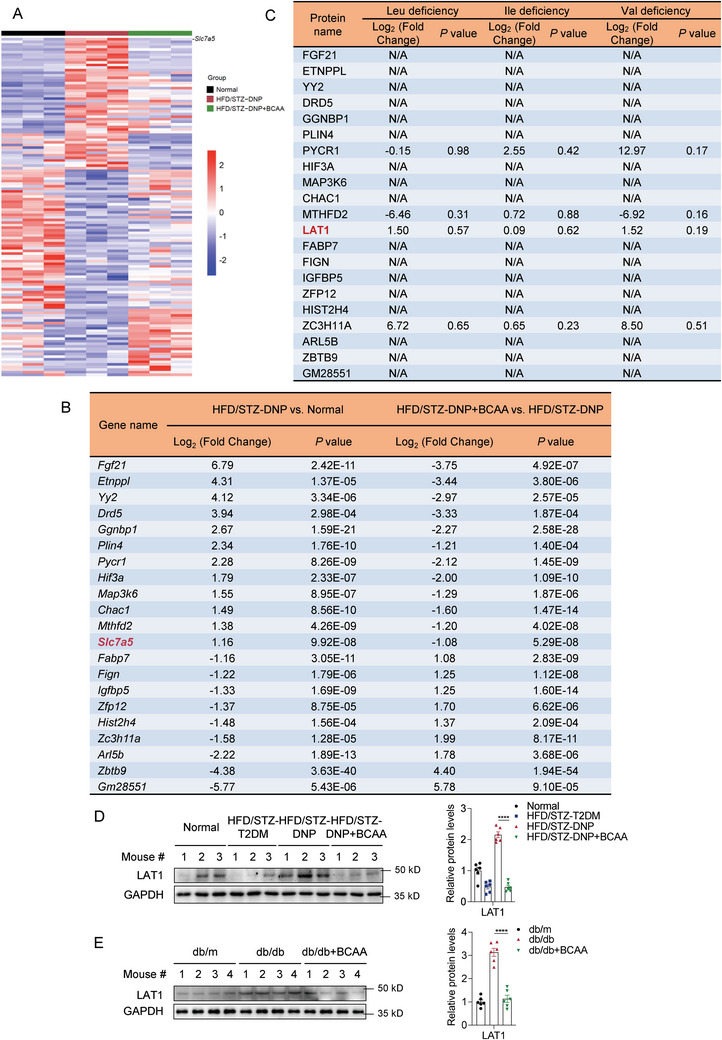
LAT1 expression levels are increased in the DRG of DNP mice and are rescued by BCAA supplementation. A) Heat maps of differentially expressed genes in DRG tissues between the Normal and HFD/STZ‐DNP groups, and between the HFD/STZ‐DNP and HFD/STZ‐DNP+BCAA groups (*n* = 3 mice per group). B) Table of 21 differentially expressed genes between the Normal and HFD/STZ‐DNP groups, which were reversed by BCAA supplementation. C) Expression levels of the 21 proteins were verified by quantitative proteomic analysis of HEK293T cells (*n* = 3 per group). D,E) Expression of LAT1 in DRG tissue samples from HFD/STZ mice (D) and db/db mice (E) after BCAA supplementation, detected by western blotting. Quantification of blots is shown on the right (*n* = 6 mice per group). Data are expressed as mean ± SEM. One‐way analysis of variance (ANOVA) was used to analyze the differences among three or more groups, followed by Bonferroni's multiple comparison test to obtain adjusted *p* values. Significance is indicated as ^ns^ *p* > 0.05, ^*^ *p* < 0.05, ^**^ *p* < 0.01, ^***^ *p* < 0.001, and ^****^ *p* < 0.0001.

This observation raises the question of how the lack of BCAA leads to the occurrence of DNP. DRG tissues of mice in the normal, HFD/STZ‐DNP, and HFD/STZ‐DNP+BCAA groups were collected for RNA sequencing analysis. *Slc7a5*, the coding gene of LAT1, ranked first in the cluster analysis (**Figure** **3**A). Among the genes exhibiting altered expression among the three groups, 12 genes were upregulated in the HFD/STZ‐DNP group compared with the normal group, and downregulated after BCAA supplementation; and 9 genes were downregulated in the HFD/STZ‐DNP group compared with the normal group, and upregulated after BCAA supplementation. The expression models of these 21 genes matched the pain behavior levels; therefore, they may be involved in the occurrence of DNP and are regulated by BCAA supplementation (Figure 3B). Subsequently, to investigate the specific changes in cellular signaling occurring due to the deficiency of each BCAA, we cultured HEK293T cells in media lacking leucine, isoleucine, or valine, and then used a proteomics approach to detect protein libraries with upregulated or downregulated expression. The 21 genes screened in the RNA sequencing results were verified using the protein library, and we found that LAT1 exhibited a consistent expression pattern in both DRG tissue RNA sequencing and cultured cell proteomics (Figure 3C). It was upregulated in both the BCAA‐deficient and HFD/STZ‐DNP groups and then decreased after BCAA supplementation.

These changes in the expression levels of LAT1 were validated using western blot in the DRG tissues of both mouse models. Compared with the HFD/STZ‐T2DM group, the expression level of LAT1 in the HFD/STZ‐DNP group was upregulated and decreased after BCAA supplementation (Figure 3D). BCAA supplementation also decreased LAT1 expression in db/db mice (Figure 3E). These results suggest that LAT1 upregulation caused by BCAA deficiency in the DRG may be a key factor in DNP development.

### 2.5 BCAA Deficiency Upregulates LAT1 Expression Through ATF4

**Figure 4:**
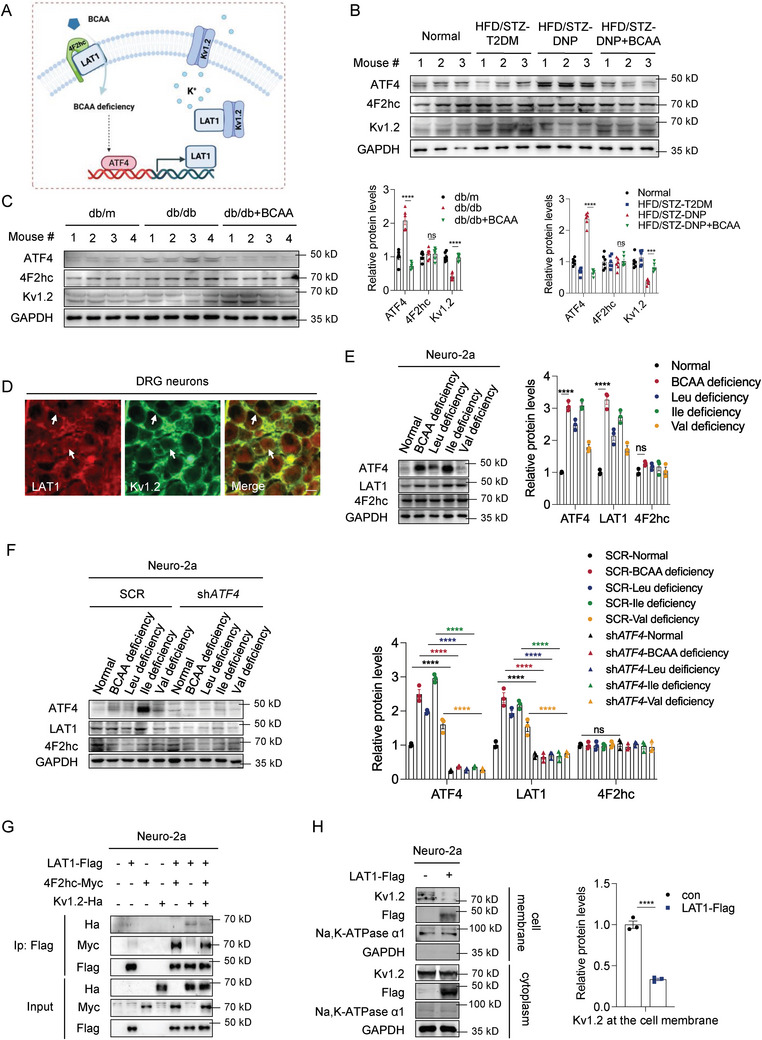
BCAA deficiency compensates for LAT1 upregulation through ATF4. A) Schematic showing that BCAA deficiency upregulates LAT1 expression by activating ATF4, and dysregulated LAT1 inhibits Kv1.2 channels. B,C) Western blot analysis of ATF4, 4F2hc, and Kv1.2 in DRG tissue samples from HFD/STZ mice (B) and db/db mice (C) after BCAA supplementation. Quantification of blots is shown below or on the right (*n* = 6 mice per group). D) Double‐labeled immunofluorescent staining showed co‐localization of LAT1 (red) with Kv1.2 (green) in the DRG neurons of naïve mice. Scale bar: 200 µm. (*n* = 3 per group). E) Western blot analysis of ATF4, LAT1, and 4F2hc in Neuro‐2a cells treated with BCAA deficiency. Quantification of blots is shown on the right (*n* = 3 per group). F) Western blot analysis of ATF4, LAT1, and 4F2hc in SCR and sh*ATF4* Neuro‐2a cells treated with BCAA deficiency. Quantification of blots is shown on the right (*n* = 3 per group). G) The interactions between Kv1.2 and LAT1, and between 4F2hc and LAT1 in Neuro‐2a cells were examined by co‐immunoprecipitation. H) Western blot analysis of Kv1.2 expression at the cell membrane in Neuro‐2a cells after overexpression of LAT1. Quantification of blots is shown on the right (*n* = 3 per group). Data are expressed as mean ± SEM. One‐way analysis of variance (ANOVA) was used to analyze the differences among three or more groups, followed by Bonferroni's multiple comparison test to obtain adjusted *p* values. Significance is indicated as ^ns^ *p* > 0.05, ^*^ *p* < 0.05, ^**^ *p* < 0.01, ^***^ *p* < 0.001, and ^****^ *p* < 0.0001.

Next, we investigated how BCAA deficiency caused LAT1 upregulation in neurons. Previous studies have shown that lower BCAA levels elicit compensatory upregulation of LAT1 by the transcription factor ATF4 in tumor cells,^[^ ^17^, ^26^, ^27^ ^]^ suggesting a potential causal relationship between BCAA deficiency and LAT1 increase. In addition, several reports found that LAT1 was able to inhibit Kv1.2 channels in mouse LM(TK‐) fibroblast cells,^[^ ^28^, ^29^, ^30^ ^]^ suggesting a potential regulatory effect of LAT1 on Kv1.2 channels. Since the Kv1.2 channel has a well‐established pathological effect on neuropathic pain, we hypothesized that BCAA deficiency in DRG neurons upregulated LAT1 levels by activating ATF4, and that dysregulated LAT1 inhibited Kv1.2 channel current density, leading to increased neuronal excitability (**Figure** **4**A).

To validate, we first performed experiments on the DRG tissues of the two mouse models. Compared to the HFD/STZ‐T2DM group, the levels of ATF4 and LAT1 were upregulated in the HFD/STZ‐DNP group and decreased after BCAA supplementation (Figures 3D and 4B). However, there was no significant change in 4F2hc levels, which was consistent with the RNA sequencing results (Figures 3B and 4B). BCAA supplementation rescued the decrease in Kv1.2 levels in the HFD/STZ‐DNP group (Figure 4B). In the db/db mouse model, BCAA supplementation downregulated the levels of ATF4 and LAT1 and upregulated the level of Kv1.2 (Figure 4C). We also investigated the expression of ATF4, LAT1, and Kv1.2 in DRG neuron subtypes and glial cells. We found that they are strongly expressed in a large majority of the NF200^+^ and NEUN^+^ DRG neurons, whereas it is poorly expressed in IB4^+^ neurons and GS^+^ glial cells (Figure S3A, Supporting Information). There was a strong co‐localization of LAT1 and Kv1.2 in the DRG tissues (Figure 4D).

Second, we demonstrated that BCAA deficiency upregulated LAT1 via the transcription factor ATF4 in Neuro‐2a cells. BCAA deficiency upregulated the mRNA and protein levels of ATF4 and LAT1 in Neuro‐2a cells, especially in isoleucine‐deficient cells (Figure 4E; Figure S3B,C, Supporting Information). However, the protein level of 4F2hc did not change (Figure 4E), indicating that BCAA deficiency specifically upregulated LAT1 in the heterodimer, suggesting that LAT1 has other biological functions besides forming a heterodimer with 4F2hc. Knockdown of ATF4 reduced the mRNA and protein levels of LAT1 (Figure 4F; Figure S3D–F, Supporting Information). Moreover, after ATF4 knockdown, BCAA deficiency no longer upregulated LAT1 protein levels, and the amplitude of LAT1 mRNA upregulation significantly decreased (Figure 4F; Figure S3D–F, Supporting Information). This illustrates the necessity of ATF4 in LAT1 upregulation in response to BCAA deficiency.

Next, our findings indicate the presence of an interaction between LAT1 and Kv1.2 in Neuro‐2a cells. Additionally, we observed that this interaction is inhibited by 4F2hc (Figure 4G). This suggests a competitive relationship between 4F2hc and Kv1.2 in their interaction with LAT1.

Finally, in Neuro‐2a cells, overexpression of LAT1 suppressed Kv1.2 protein levels at the cell membrane (Figure 4H). These results collectively demonstrate that BCAA deficiency leads to the upregulation of LAT1 through ATF4, and confirm the potential role of LAT1 in regulating Kv1.2 channels.

### 2.6 BCAA Deficiency Increases Excitability of Primary DRG Neurons

**Figure 5:**
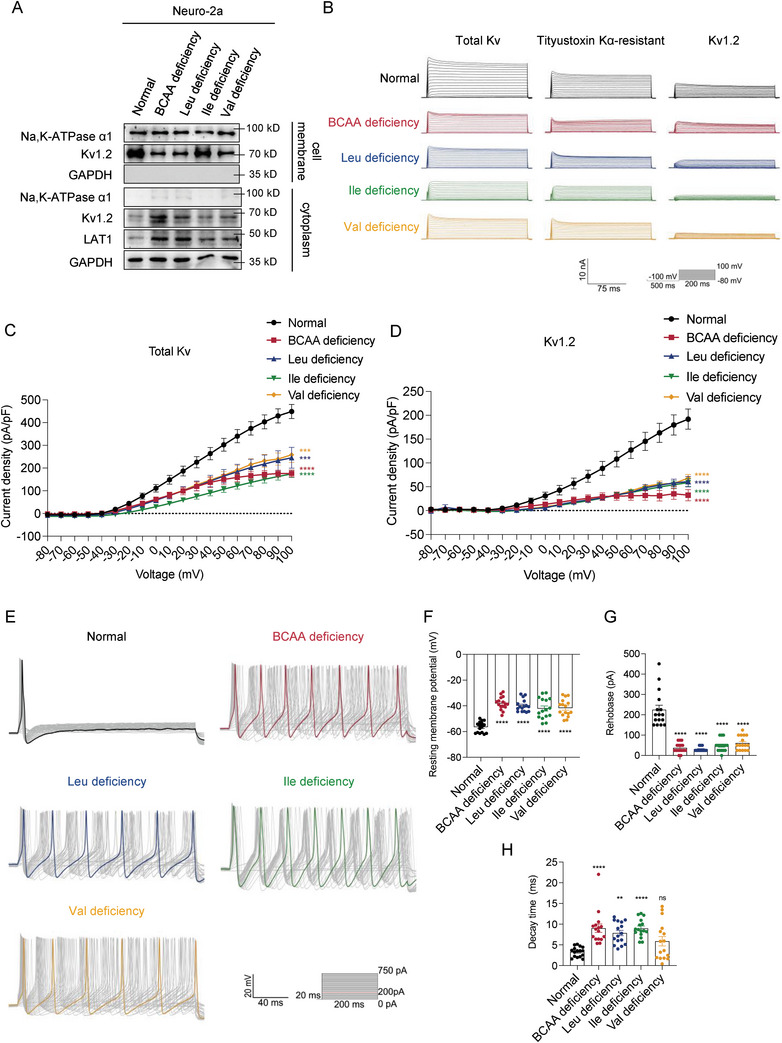
BCAA deficiency increases the excitability of primary DRG neurons. A) Western blot analysis of Kv1.2 expression at the cell membrane in Neuro‐2a cells treated with BCAA deficiency (*n* = 3 per group). B) Representative original traces of Kv currents in primary DRG neurons treated with BCAA deficiency. C,D) Current density of total Kv currents (C) and Kv 1.2 currents (D) in primary DRG neurons treated with BCAA deficiency (*n* = 8 per group). E) Representative action potential traces of primary DRG neurons treated with BCAA deficiency. F–H) Resting membrane potential (F), action potential rheobase (G), and decay time (H) of primary DRG neurons treated with BCAA deficiency (*n* = 16 per group). Data are expressed as mean ± SEM. One‐way analysis of variance (ANOVA) was used to analyze the differences among three or more groups, followed by Bonferroni's multiple comparison test to obtain adjusted *p* values. Significance is indicated as ^ns^ *p* > 0.05, ^*^ *p* < 0.05, ^**^ *p* < 0.01, ^***^ *p* < 0.001, and ^****^ *p* < 0.0001.

Next, we investigated whether BCAA deficiency inhibited Kv1.2 channels and increased the excitability of DRG neurons. LAT1 decreases the expression of the mature, fully glycosylated form of Kv1.2, which is mainly expressed at the cell surface.^[^ ^28^, ^29^, ^31^ ^]^ In accordance with these findings, we observed that BCAA deficiency reduced the protein levels of Kv1.2 at the cell membrane in Neuro‐2a cells (**Figure** **5**A). We examined the Kv currents in acutely isolated small L3‐L5 DRG neurons treated with BCAA deficiency. The Kv currents were also recorded after treatment with Tityustoxin‐Kα, a biologically active Kv1.2 channel blocker. The currents of the Kv1.2 channels were obtained by subtracting the two currents (Figure 5B). BCAA deficiency significantly reduced the peak amplitudes of both total Kv and Kv1.2 currents (Figure 5C,D). Among them, isoleucine deficiency reduced Kv currents the most significantly.

We further analyzed the neuronal excitability in small L3‐L5 DRG neurons using whole‐cell current‐clamp recordings (Figure 5E). The analysis revealed a significantly decreased resting membrane potential (Figure 5F), a significantly lower rheobase (Figure 5G), and a significantly longer decay time (Figure 5H) in DRG neurons treated with BCAA deficiency compared to the normal group, consistent with the current reduction in Kv1.2 channels. Overall, these results demonstrate that BCAA deficiency inhibits Kv1.2 channels and increases the excitability of DRG neurons, which in turn contributes to the development of neuropathy.

### 2.7 BCH Rescued the Increased Excitability of Primary DRG Neurons Caused by BCAA Deficiency

Although BCAA supplementation can help slow the onset of diabetic neuropathy, BCAA may have harmful effects on the development of diabetes. Therefore, we attempted to develop new potential treatment methods by intervening in the downstream pathways responsible for BCAA‐deficiency‐induced diabetic neuropathy. We explored whether the inhibition of upregulated LAT1 alleviated DNP symptoms in mice. Previous studies have shown that loss of LAT1 causes various nervous system lesions,^[^ ^13^, ^14^ ^]^ and some LAT1 inhibitors have been widely reported in cancer research.^[^ ^26^, ^27^ ^]^ Therefore, we used an inhibitor rather than a specific knockout to inhibit LAT1. Considering that most of these are competitive inhibitors which, according to existing reports, upregulate LAT1 expression levels,^[^ ^32^ ^]^ only BCH downregulates ATF4 protein levels;^[^ ^33^ ^]^ therefore, we decided to use BCH to reduce the protein levels of LAT1.

**Figure 6:**
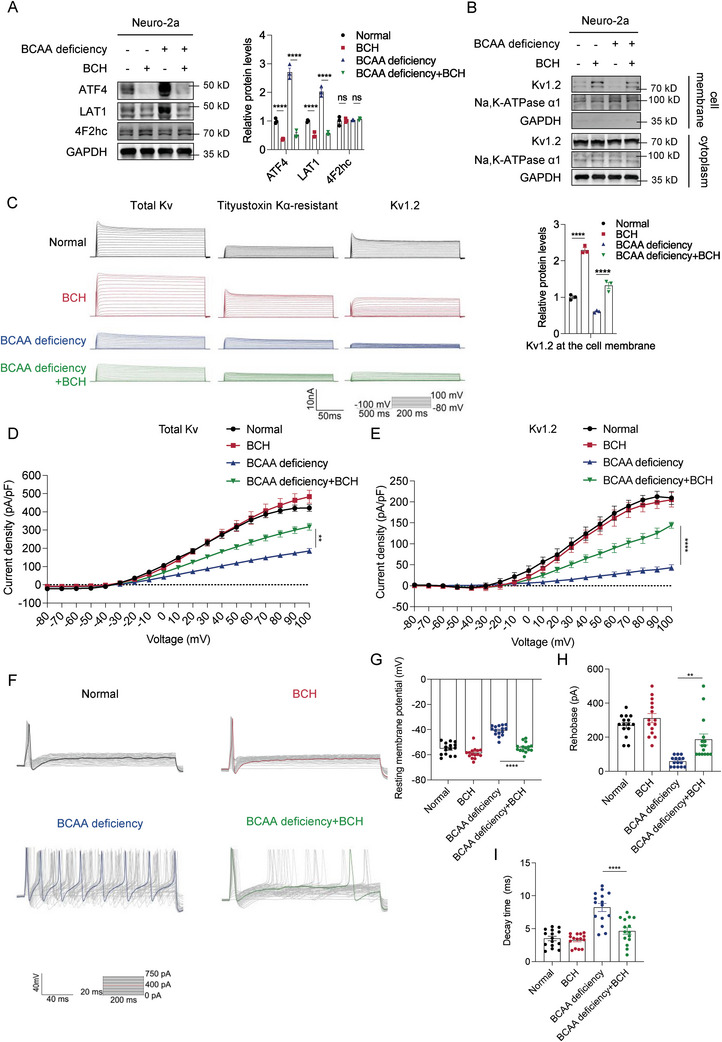
BCH rescued the increased excitability of primary DRG neurons caused by BCAA deficiency. A) Western blot analysis of ATF4, LAT1, and 4F2hc expression in Neuro‐2a cells treated with BCAA deficiency or BCH. Quantification of blots is shown on the right (*n* = 3 per group). B) Western blot analysis of Kv1.2 expression at the cell membrane in Neuro‐2a cells treated with BCAA deficiency or BCH. Quantification of blots is shown below (*n* = 3 per group). C) Representative original traces of Kv currents in primary DRG neurons treated with BCAA deficiency or BCH. D,E) Current density of total Kv currents (D) and Kv 1.2 currents (E) in primary DRG neurons treated with BCAA deficiency or BCH (*n* = 12 per group). F) Representative action potential traces of primary DRG neurons treated with BCAA deficiency or BCH. G–I) Resting membrane potential (G), action potential rheobase (H), and decay time (I) of primary DRG neurons treated with BCAA deficiency or BCH (*n* = 15 per group). Data are expressed as mean ± SEM. One‐way analysis of variance (ANOVA) was used to analyze the differences among three or more groups, followed by Bonferroni's multiple comparison test to obtain adjusted *p* values. Significance is indicated as ^ns^ *p* > 0.05, ^*^ *p* < 0.05, ^**^ *p* < 0.01, ^***^ *p* < 0.001, and ^****^ *p* < 0.0001.

First, we examined the effect of BCH treatment on LAT1 protein levels in Neuro‐2a cells. BCH treatment significantly suppressed the protein levels of ATF4 and LAT1 upregulated by BCAA deficiency in Neuro‐2a cells (**Figure** **6**A). Kv1.2 protein levels at the cell membrane were also upregulated after BCH treatment (Figure 6B).

Next, we investigated whether BCH treatment rescued the increased excitability of primary DRG neurons caused by BCAA deficiency. We examined the Kv currents in acutely isolated small L3‐L5 DRG neurons treated with BCH. BCH treatment significantly increased the peak amplitudes of both total Kv and Kv1.2 currents inhibited by BCAA deficiency (Figure 6C–E). We further analyzed the neuronal excitability in DRG neurons (Figure 6F). BCH treatment rescued resting membrane potential (Figure 6G), rheobase (Figure 6H), and decay time (Figure 6I) altered by BCAA deficiency. Overall, these results demonstrate that BCH rescued the increased excitability of primary DRG neurons caused by BCAA deficiency.

### 2.8 LAT1 Inhibitor BCH Alleviates the Symptoms of DNP in Mice

**Figure 7:**
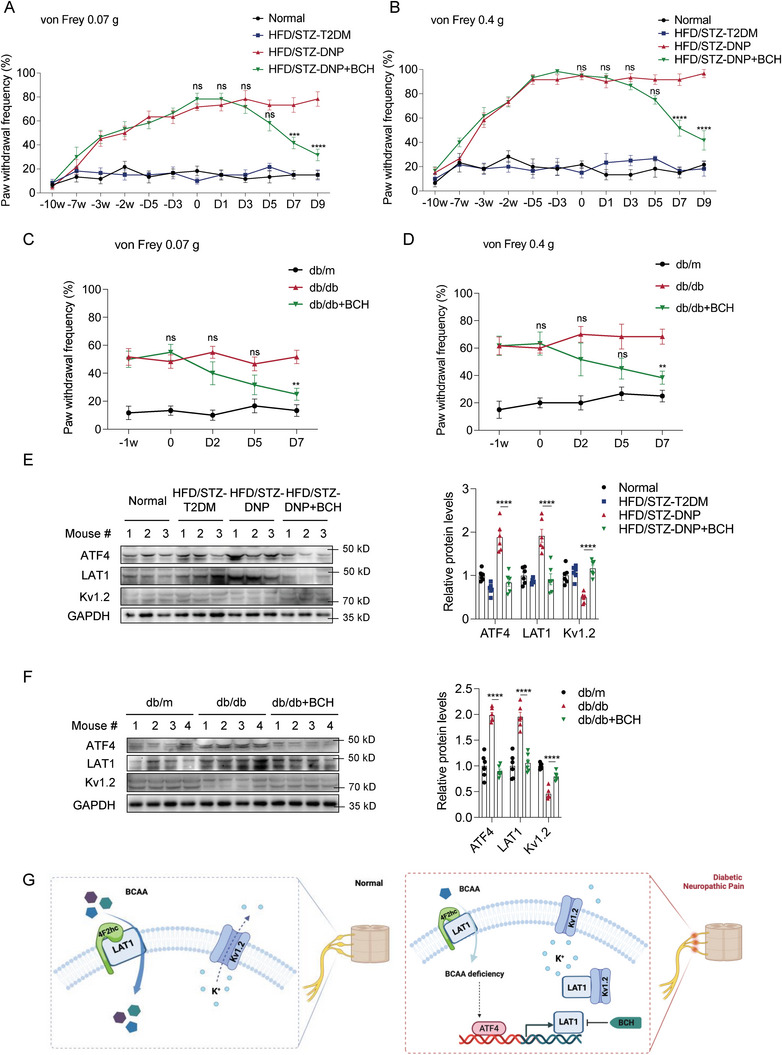
Short‐term intraperitoneal injection of BCH alleviates mechanical allodynia in HFD/STZ mice and db/db mice. A,B) The paw withdrawal frequencies (PWF) to 0.07 g (A) and 0.4 g (B) von Frey filaments were detected on the different days during the HFD/STZ mouse model establishment and short‐term intraperitoneal injection of BCH (*n* = 6 mice per group). C,D) The paw withdrawal frequencies (PWF) to 0.07 g (C) and 0.4 g (D) von Frey filaments were detected on different days during the short‐term intraperitoneal injection of BCH in db/db mice (*n* = 6 mice per group). E,F) Western blot analysis of ATF4, LAT1, and Kv1.2 in the DRG tissue samples from HFD/STZ mice (E) and db/db mice (F) after short‐term intraperitoneal injection of BCH. Quantification of blots is shown on the right (*n* = 6 mice per group). G) Schematic showing that BCAA deficiency in the DRG inhibits the Kv1.2 channels by abnormally upregulating LAT1 expression, thereby increasing the excitability of neurons and contributing to the occurrence of DNP. Data are expressed as mean ± SEM. One‐way analysis of variance (ANOVA) was used to analyze the differences among three or more groups, followed by Bonferroni's multiple comparison test to obtain adjusted *p* values. Differences between the HFD/STZ‐DNP and HFD/STZ‐DNP+BCH groups, and between the db/db and db/db+BCH groups are shown. Significance is indicated as ^ns^ *p* > 0.05, ^*^ *p* < 0.05, ^**^ *p* < 0.01, ^***^ *p* < 0.001, and ^****^ *p* < 0.0001.

Next, we administered BCH to both mouse models by short‐term intraperitoneal injection. Mechanical pain tests showed that intraperitoneal administration of BCH significantly attenuated mechanical allodynia, demonstrated by decreased paw withdrawal frequencies in response to low (0.07 g) and medium (0.4 g) force von Frey filaments, in HFD/STZ (**Figure** **7**A,B) and db/db (Figure 7C,D) mouse models. However, the effect of BCH was weaker than that of direct BCAA supplementation in both mouse models. Similar to BCAA supplementation, BCH decreased the levels of ATF4 and LAT1 and increased the level of Kv1.2 in the DRG tissues in both mouse models (Figure 7E,F). These results confirm that BCAA‐deficiency‐induced LAT1 activation contributes to the onset of DNP and that the short‐term use of BCH targeting LAT1 is a good choice to treat DNP symptoms in mice.

## 3 Discussion

We focused on the differences in metabolites between DNP and T2DM and found that a decrease in BCAA levels was significantly associated with the occurrence of DNP in patients with T2DM. BCAA deficiency aggravated, whereas BCAA supplementation significantly attenuates mechanical allodynia in mouse models. These findings provide fresh insights into metabolic differences between DNP and T2DM.

BCAA are essential amino acids and nutrient signals that exert direct and indirect effects.^[^ ^10^ ^]^ Whether increased BCAA levels are biomarkers, pathogenic agents, or both, is still being deliberated.^[^ ^11^ ^]^ The role of BCAA in the nervous system has attracted considerable attention. Recent studies have shown that intraventricular injection of BCAA alleviates a range of behavioral abnormalities in mice and that BCAA are essential for perinatal neuronal metabolic states and survival.^[^ ^13^, ^14^ ^]^ A study on the synergistic effect of exercise and BCAA on brain structure and function found that exercising mice only fed dietary BCAA showed improvements in spatial learning and memory.^[^ ^34^ ^]^ In conclusion, whether BCAA or BCAA supplementation is harmful, beneficial, or both, requires further research. Perhaps, the role of BCAA needs to be viewed dialectically at different stages of the disease, and in different tissues.

The collaborative switching of the two biological functions of LAT1: the uptake of BCAA and the inhibition of Kv1.2 is another issue that we focused on. LAT1 is often of interest because of its overexpression in tumor cells, which requires large amounts of amino acids.^[^ ^26^, ^27^ ^]^ There is also evidence that LAT1 is involved in pain,^[^ ^35^ ^]^ such as increased LAT1 levels in DRG and spinal cord of spared nerve injury mice.^[^ ^36^ ^]^ Although some reports have provided evidence that LAT1 inhibits Kv1.2 channel current density,^[^ ^28^, ^29^, ^30^ ^]^ its physiological significance is not fully understood. No direct studies have been conducted on the relationship between LAT1 and neuropathic pain. Our study is the first to unify the two biological functions of LAT1 in DRG tissues, which require BCAA and express Kv1.2. Under physiological conditions, LAT1 plays a role in BCAA uptake; and abnormally high LAT1 expression due to BCAA deficiency inhibits Kv1.2 channels. In addition, the antitumor drug BCH used in the treatment of DNP in mice has also provided good insight, and targeting LAT1 may be the direction of DNP treatment worth studying in the future. Whether LAT1 also plays an important role in neuropathic pain and affects other ion channels requires further investigation.

This article focuses on the role of BCAA deficiency and abnormal upregulation of LAT1 in the pathogenesis of DNP, but there must be other metabolites or proteins that play key roles. After a PubMed search, there is currently no clue that other genes listed in Figure 3B are directly associated with both BCAA and neuropathic pain. More comprehensive metabolomics combined with proteomics and transcriptome analysis of clinical samples will help to find more clues.

Current DNP mouse models tend to utilize existing mouse models of type 1 and type 2 diabetes.^[^ ^37^, ^38^, ^39^ ^]^ On this basis, the neuropathy phenotype was observed. Of the many behavioral tests available, touch and thermal stimuli most closely reflect the aspects of clinical Quantitative Sensory Testing and do not require animal restraint.^[^ ^38^ ^]^ The occurrence and extent of DNP may vary depending on the mouse model, mouse strain, mouse age, and the severity of insulin deficiency and/or hyperglycemia.^[^ ^38^ ^]^ In the HFD/STZ mouse model, some with symptoms of DNP and others without, this study found that upregulation of LAT1 expression is one of the reasons. However, the reasons for such differences in mice with the same genetic background, modeling method, and feeding environment need to be further studied. All the db/db mice tested in this study showed DNP symptoms, despite individual differences. Differences were also observed between the HFD/STZ and db/db mouse models in response to BCAA and BCH treatment. The metabolic phenotype must also be determined to fully characterize DNP in mouse models. It seems insufficient to consider basic metabolic indicators, such as body weight, FBG, and glucose tolerance. Mouse models with glycemic control but persistent diabetic complications, such as DNP have not been developed to mimic the real situation in humans. There is still a long way to go to establish an ideal mouse model of DNP, which should be complemented by an in‐depth understanding of DNP pathogenesis.

Our study had several limitations. First, the plasma BCAA levels were significantly lower in patients with DNP than in those with T2DM; however, the potential reasons for this are not clear. This phenomenon may be due to dietary habits, abnormal BCAA metabolism in other tissues such as the liver and muscle, or both. Finally, leucine, isoleucine, and valine are commonly studied together because of their structural similarities. Consistent with clinical findings (Table 1), isoleucine was found to be more important during DNP development in both Neuro‐2a cells (Figure 4E,F; Figure S3B–F, Supporting Information) and primary DRG neurons (Figure 5C,H). The effect of the valine deficiency was weaker. The differences among the three BCAA were beyond the scope of this study but worthy of attention.

In summary, we found that decreased BCAA levels were significantly associated with DNP. Mechanistically, BCAA deficiency in the DRG led to LAT1 upregulation via the ATF4 pathway. Abnormally upregulated LAT1 inhibits Kv1.2 channels, thereby increasing neuronal excitability and causing neuropathy (Figure 7G). Both BCAA supplementation and intraperitoneal injection of BCH improved DNP symptoms in mice.

## 4 Experimental Section

### Study Participants

The study design was approved and supervised by Shanghai Tenth People's Hospital, following the criteria established by the Declaration of Helsinki. All participants or their authorized representatives provided written informed consent prior to study‐related procedures. This study included adult participants with and without T2DM. Fasting plasma samples were obtained from the participants in the morning at Shanghai Tenth People's Hospital from July 2021 to July 2023. A total of 194 patients were screened in this study. The patients included 73 T2DM patients without neuropathy and 81 with DNP. Thirty healthy individuals were recruited as controls. Individuals with a history of cardiovascular disease or metabolic disorders, including uncontrolled hypertension or hyperthyroidism, were excluded, as were those with abnormal liver or kidney function. The inclusion criteria for patients with DNP were as follows: 1) a definite history of type 2 diabetes mellitus; 2) neuropathy occurring at or after the diagnosis of diabetes; and 3) clinical symptoms of neuropathy pain, which may be accompanied by numbness, paresthesia, etc. When the clinical diagnosis was in doubt, a neuroelectrophysiological examination was performed. Patients with numbness or chills but no pain were excluded, as were patients with other causes of neuropathy pain, including neurotoxic drugs (such as chemotherapy drugs), vitamin B12 deficiency, cervical and lumbar diseases, cerebral infarction, chronic inflammatory demyelinating neuropathy, hereditary neuropathy and vasculitis, infection (such as acquired immunodeficiency syndrome), and metabolic poisons caused by renal insufficiency. Age‐ and sex‐matched individuals with T2DM but without neuropathy were included in the T2DM group. The clinical characteristics of the study participants are presented in Table S1 (Supporting Information). The medication use of the participants is shown in Table S2 (Supporting Information).

### Nuclear Magnetic Resonance Analysis of Metabolite

Nuclear magnetic resonance (NMR) analysis was adapted from published methods.^[^ ^21^, ^22^, ^23^ ^]^ For serum samples, metabolites in 200 µL serum samples were extracted by adding 800 µL prechilled methanol. Supernatants were collected after centrifugation (12000 × *g*, 4 °C) for 15 min. The extraction was repeated twice, and the supernatants were combined and lyophilized after vacuum drying to remove methanol and water. The extracted metabolites were redissolved in a 570 µL phosphate buffer (0.15 m, pH 7.4, 80% D_2_O, TSP 0.2915 mm) vortex. The resulting solution was centrifuged at 16000 × *g* (4 °C) for 10 min before the 530 µL supernatant was transferred into NMR tubes for analysis. All the 1D ^1^H NMR spectra were acquired at 298 K on a Bruker Advance III 600 MHz NMR spectrometer (600.13 MHz for proton frequency) equipped with a quaternary cryogenic inverse probe (Bruker Biospin, Germany) using the first increment of the gradient selected NOESY pulse sequence (NOESYGPPR1D: recycle delay‐G1‐90^0^‐T1‐90^0^‐tm‐G2‐90^0^‐acquisition). In total, 64 transients were collected into 32 k data points with a spectral width of 20 ppm for each sample, and the total relaxation delay time was 26 s. All NMR spectra were processed using the software package TOPSPIN (V3.6.0, Bruker Biospin, Germany). For ^1^H NMR spectra, an exponential window function was employed with a line broadening factor of 1 Hz and zero–filled to 128 K before Fourier transformation. The characteristic and least‐overlapping NMR signals were used to calculate the absolute concentration of metabolites with the known concentration of TSP.

### Animal Models

All animal procedures were performed according to the guidelines of Shanghai Tenth People's Hospital. All mice were housed in polycarbonate cages and provided free access to food and water under a 12 h light‐dark cycle.

Two different T2DM models were used: 1) A high‐fat diet/low‐dose streptozotocin (HFD/STZ)‐induced T2DM mouse model; and 2) A leptin receptor‐deficient diabetic (db/db) mouse model. For the HFD/STZ mouse model, 8‐week‐old male C57BL/6 mice were obtained from GemPharmatech Co., Ltd. (Jiangsu, China) and randomly divided into normal and experimental groups. The normal group was fed a regular diet (10 kcal% fat, D12450J, Wuxi Fanbo Biotechnology Co., Ltd., China). To induce insulin resistance and obesity, the experimental group was fed an HFD (60 kcal% fat, D12492, same as above) for 8 weeks and received an intraperitoneal injection of STZ (100 mg kg^−1^, MS1601, Shanghai Maokang Biotechnology Co., Ltd, China). Two weeks after STZ injection, mice with FBG higher than 16.7 mmol L^−1^ were included in the formal experiment. The 8‐week‐old male C57BLKS/J db/db mice obtained from Junke Bioengineering Co., Ltd. (Nanjing, China) were used as the second experimental group, and C57BLKS/J db/m mice were used as the corresponding control group. To ensure consistency, both groups were fed a standard laboratory diet. Body weight, FBG levels, and insulin resistance were regularly measured to monitor T2DM progression.

For BCAA deficiency and supplementation feeding, all mice were fed an L‐amino acid rodent diet (FB‐A10021B, containing isoleucine 8 g kg^−1^, leucine 12 g kg^−1^, valine 8 g kg^−1^, same as above) for adaptation one week in advance after the T2DM models were successfully established. The BCAA deficiency group was fed with halved BCAA content (FB‐A10022, containing isoleucine 4 g kg^−1^, leucine 6 g kg^−1^, valine 4 g kg^−1^, same as above). The BCAA supplementation groups were fed a diet with double BCAA content (FB‐A10023, containing isoleucine 16 g kg^−1^, leucine 24 g kg^−1^, valine 16 g kg^−1^, same as above). Mice in all other groups continued to be fed the FB‐A10021B diets.^[^ ^40^, ^41^ ^]^ Specific dietary information is presented in Table S3 (Supporting Information).

For BCH treatment, mice were administered daily intraperitoneal injections of BCH (HY‐108540, MedChemExpress) at a dose of 240 mg kg^−1^ for 1 week at appropriate time points. Blood serum and tissues were collected at specific time points during the experiment.

### Behavioral Tests

Behavioral tests were performed as published methods.^[^ ^42^, ^43^ ^]^ All mice were habituated to the testing environment daily for 3 days before baseline testing. Before testing, animals were allowed to acclimate to the environment for 1 h in a temperature‐controlled and noise‐free room (24 ± 2 °C). The evoked behavioral tests, including mechanical, heat, and cold tests, were carried out in sequential order at 1 h intervals.

First, paw withdrawal frequencies (PWF) in response to mechanical stimuli were measured. Each mouse was placed in a plastic chamber on a metal mesh floor and allowed to acclimate for 30 min. Two calibrated von Frey filaments (0.07 and 0.4 g, Aesthesio, Danmic Global) were used to stimulate the hind paw for ≈1 s and each stimulation was repeated 10 times to both hind paws with 5 min intervals. A quick withdrawal of the paw was considered a positive response. The number of positive responses among the 10 applications was recorded as the percentage of withdrawal frequency ([number of paw withdrawals per 10 trials] × 100 = % response frequency). The symptoms of DNP were detected using mechanical pain measurements during the entire process of model establishment.

Paw withdrawal latencies in response to noxious heat stimuli were measured using an IITC plantar analgesia meter (IITC Life Science Inc.). Briefly, mice were placed in individual Plexiglas chambers on a glass plate. A beam of light was emitted from a hole in the lightbox and applied to the middle of the plantar surface of each hind paw. The light beam was turned off when the mouse withdrew its foot. The paw withdrawal latency was defined as the length of time between the start of the light beam and the end of the light beam. Each test was repeated five times at 10‐min intervals for the paw on each side. A cut‐off time of 20 s was used to avoid tissue damage to the hind paw.

Paw withdrawal latencies to noxious cold (0 °C) were examined. The mice were placed in individual Plexiglas chambers on a cold aluminum plate, the temperature of which was continuously monitored using a thermometer (Ugo Basile, Italy). The paw withdrawal latency was recorded as the duration between placement on the plate and the first sign of jumping and/or flinching. Each test was repeated five times at 10‐min intervals for the paw on the ipsilateral side. A cut‐off time of 20 s was used to avoid tissue damage to the hind paw.

### BCAA Assay Kit

Total BCAA levels were measured using Branched Chain Amino Acid Assay Kit/BCAA Assay Kit (ab83374), following the assay protocol. Briefly, 20 µL plasma was added to a 96‐well plate, and then BCAA assay buffer (30 µL per well) was added. Per well‐containing leucine standard or test samples, 50 µL reaction mix containing 46 µL assay buffer, 2 µL enzyme mix, and 2 µL developer solution 3 per substrate mix was added and mixed well. After incubation in the dark for 30 min at room temperature, the optical density was measured at 450 nm using a microplate reader. Data were background‐corrected, and standard curves were plotted for each sample to calculate BCAA concentrations in the samples.

### Glucose and Insulin Tolerance Tests

Assays were performed in male mice following BCAA supplementation. For GTTs, mice were intraperitoneally injected with glucose (1 g kg^−1^) after 16 h of fasting, and blood was sampled at 0, 15, 30, 60, and 120 min after glucose injection. For ITTs, mice were intraperitoneally injected with 0.4‐0.5 units kg^−1^ of insulin after 6 h of fasting, and blood was sampled at 0, 15, 30, 60, and 120 min after insulin injection.

### RNA Sequencing (RNA‐Seq)

Library construction and sequencing were performed by the Shanghai Bohao Biotechnology Corporation. Three groups of mice were used, each with three replicates. Total RNA was isolated from mouse L3‐L5 DRG neurons using a RNeasy Mini Kit (Qiagen, Hilden, Germany). Paired‐end libraries were synthesized by using the TruSeq RNA Sample Preparation Kit (Illumina) following TruSeq RNA Sample Preparation Guide. Briefly, poly (A)‐containing mRNA was purified using poly (T) oligo‐attached magnetic beads. Purified libraries were quantified by Qubit 2.0 Fluorometer (Life Technologies) and validated by Agilent 2100 bioanalyzer (Agilent Technologies) to confirm the insert size and calculate the mole concentration. Clusters were generated using cBot with the library diluted to 10 pmol L^−1^ and sequenced on an Illumina HiSeq X‐ten (Illumina).

Sequencing raw reads were pre‐processed by filtering rRNA reads, sequencing adapters, short fragment reads, and other low‐quality reads. Hisat2 (version 2.0.4) was used to map the cleaned reads to the human GRCh38 reference genome with two mismatches. After genome mapping, StringTie (version1.3.0) was run with reference annotations to generate FPKM values for known gene models. Differentially expressed genes were identified using edgeR software. The *P* value significance threshold for multiple tests was set using the false discovery rate (FDR). The fold changes were also estimated according to the FPKM in each sample. The differentially expressed genes were selected using the following filter criteria: FDR ≤ 0.05 and |log_2_fold change| ≥ 0.

### Label‐Free Quantitative Proteomics

Quantitative proteomic analysis of cell samples was performed using the Proteome Platform of the Human Phenome Institute (Fudan University, China). HEK293T cells were treated with isoleucine, leucine, and valine deficiency in triplicate per group. Samples from the different groups were collected for label‐free quantitative proteomic analysis (EASY‐nLC 1200, Q Exactive HFX, Thermo Fisher Scientific). Firmiana was used for data analysis. ABQ was used to quantify abundance, and FOT was used to represent the protein quantification value and protein abundance after data normalization. Next, differentially expressed genes were analyzed, and fold changes and *P* values were calculated.

### Immunohistochemistry

Mice were anesthetized and perfused with 4% PFA in PBS. L3/L4 DRGs were dissected and post‐fixed in PFA on ice for 2 h and then cryoprotected in 30% sucrose for 72 h at 4 °C. The DRGs were sectioned at 15‐µm thickness. The slides were treated with 5% normal goat serum and 0.3% Triton X‐100 and then incubated with primary antibodies at 4 °C overnight. The next day, slides were washed with TBST 3 times and then incubated with species‐appropriate Cy3‐conjugated secondary antibody (1:500, Jackson ImmunoResearch) or with FITC‐labeled Avidin D (1:200, Sigma) for 2 h at room temperature. The sections were finally washed and mounted using VectaMount permanent mounting medium (Vector Laboratories, Burlingame, CA). Images were taken with a Leica DMI4000 fluorescence microscope (Leica) with a DFC365 FX camera (Leica) and analyzed using Image J software. The primary antibodies used in these studies were:anti‐ATF4 (1:100, 10835‐1‐AP, Proteintech), anti‐LAT1 (1:100, #5347, CST), anti‐Kv1.2 (1:1000, ab192758, abcam), anti‐Kv1.2 (1:100, 14235‐1‐AP, Proteintech), anti‐NEUN (1:3000, Sigma‐Aldrich, ABN90), anti‐NF200 (1:100, Sigma‐Aldrich, #N4142), anti‐isolectin B4 (1:250, Thermo Fisher Scientific, I21411), anti‐CGRP (1:100, Abcam, ab81887), anti‐glutamine synthetase (1:100, Millipore, MAB302).

### Cell Culture and Treatment

HEK293T cells and Neuro‐2a cells were cultured in Dulbecco's Modified Eagle's Medium (DMEM) (Gibico) supplemented with 10% fetal bovine serum (FBS) (Gibico), 100 units mL^−1^ penicillin (Invitrogen) and 100 mg mL^−1^ streptomycin (Invitrogen).

For BCAA deficiency experiments, cells were cultured in a custom‐made BCAA‐deprivation medium (Shanghai Zongji Biotechnology, Inc., China) with the remaining components and concentrations identical to those in the abovementioned DMEM. The leucine‐deficient medium was obtained by adding isoleucine and valine, and the complete medium was obtained by adding all three BCAA. To study BCAA deficiency effects, cells at the logarithmic growth stage were seeded into 6‐well plates and cultured for 24 h in the incubator (37 °C, 5% CO_2_).

For BCH treatment experiments, the concentration of BCH (HY‐108540, MedChemExpress) was 10 mm and the treatment time was 24 h.

### ATF4 Stable Knockdown Cell Line

HEK293T cells were co‐transfected with psPAX2, pMD2.G, and pLKO.1‐ATF4. Transfected cells were cultured in DMEM containing 10% FBS for 12 h. After 48 h of transfection, the lentivirus supernatant was collected and used to infect Neuro‐2a cells at a density of 10% confluence in 90 mm diameter dishes. Subsequently, 8 µg mL^−1^ polybrene (C0351, Beyotime Biotechnology, China) was used to increase infection efficiency, and 24 h after the first infection, cells were re‐infected for 24 h. After 48 h of the first infection, cells were selected using 1 µg mL^−1^ puromycin (ST551, Beyotime Biotechnology, China). Mouse ATF4 shRNA was cloned into the AgeI and EcoRI restriction sites of the pLKO.1 vector. The target sequence was CCTCTAGTCCAAGAGACTAAT. The sequences of used oligo were: sh*ATF4* forward, 5′‐CCGGCCTCTAGTCCAAGAGACTAATCTCGAGATTAGTCTCTTGGACTAGAGGTTTTTG‐3′; sh*ATF4* reverse, 5′‐AATTCAAAAACCTCTAGTCCAAGAGACTAATCTCGAGATTAGTCTCTTGGACTAGAGG‐3′.

### Plasmids Constructs and Transfection

Whole mouse LAT1, 4F2hc, and Kv1.2 were amplified from DRG tissue cDNA and cloned into the XhoI and EcoRI restriction sites of the pcDNA3.1‐Flag/Myc/Ha vector using Phanta Max Super‐Fidelity DNA Polymerase (P505, Vazyme, China) and the ClonExpress II One Step Cloning Kit (C112, Vazyme, China). Each plasmid was transfected using Lipo8000 Transfection Reagent (C0533, Beyotime Biotechnology, China) according to the manufacturer's instructions. The primers used were as follows:

LAT1: forward, 5′‐AACGGGCCCTCTAGACTCGAGATGGCGGTCGCGGGCGCC‐3′;

reverse, 5′‐TAGTCCAGTGTGGTGGAATTCAGTCTCCTGAGGTACCACCTGCA‐3′;

4F2hc: forward, 5′‐AACGGGCCCTCTAGACTCGAGATGGATCCTGAACCTACTGAACACT‐3′;

reverse, 5′‐TAGTCCAGTGTGGTGGAATTCGGCCACAAAGGGGAACTGTAA‐3′;

Kv1.2: forward, 5′‐AACGGGCCCTCTAGACTCGAGATGACAGTGGCTACCGGAGACC‐3′;

reverse, 5′‐TAGTCCAGTGTGGTGGAATTCGACATCAGTTAACATTTTGGTAATATTCA‐3′.

### Co‐Immunoprecipitation

For Co‐Immunoprecipitation, the Neuro‐2a cells were lysed with 0.5% NP‐40 buffer (50 mm Tris‐HCl (pH 7.5), 150 mm NaCl, 0.5% NP‐40, 1 µg mL^−1^ aprotinin, 1 µg mL^−1^ leupeptin, 1 µg mL^−1^ pepstatin and 1 mm PMSF). Cell lysates were incubated with Anti‐Flag Affinity Gel (HY‐K0217, MedChemExpress) for 12 h at 4 °C. The lysates were then cleared by centrifugation at 15000 × *g* for 20 min (4 °C). The binding complexes were washed with 0.5% NP‐40 buffer and mixed with loading buffer for western blotting.

### Cell Surface Biotinylation and Isolation

Cell surface biotinylation and isolation were performed as previously described.^[^ ^44^ ^]^ Briefly, Neuro‐2a cells were washed twice in ice‐cold PBS and subsequently incubated in 1 mL of 1 mm Sulfo‐NHS‐LC‐Biotin (A8003, APExBIO) in PBS for 30 min on ice. The cells were then washed in quenching buffer (100 mm glycine in PBS) and incubated in 1 mL quenching buffer for 15 min on ice. The cells were washed twice with PBS and then lysed in 300 µL of RIPA Lysis Buffer (P0013B, Beyotime Biotechnology, China) and protease inhibitor (same as above). The lysates were incubated for 20 min on ice and sonicated for 20 s. Finally, the lysate was centrifuged at 4 °C for 10 min at 15000 × *g*. Supernatants were incubated with 40 µL of Streptavidin Agarose beads (P2151, Beyotime Biotechnology, China) with constant rocking for 3 h at RT. The samples were washed three times with PBS and mixed with the loading buffer for western blotting.

### Western Blotting

L3‐L5 DRG neurons or cultured cells were homogenized and lysed with 0.5% NP‐40 buffer (same as above) for 30 min on ice. The lysates were centrifuged at 4 °C for 20 min at 15000 × *g*, and the supernatants were used as whole‐cell extracts. Protein samples were subjected to western blotting according to standard procedures. Detection was performed by measuring chemiluminescence using an ECL Plus Western Blotting Detection System on a Typhoon FLA 9500 (both GE Healthcare). The following primary antibodies were used for western blot analysis: anti‐ATF4 (1:1000, 10835‐1‐AP, Proteintech), anti‐LAT1 (1:1000, #5347, CST), anti‐Kv1.2 (1:1000, ab192758, abcam), anti‐GAPDH (1:5000, AC001, ABclonal), anti‐Na,K‐ATPase α1 (1:1000, #3010, CST), anti‐Flag (1:3000, M20008H, Abmart), anti‐Ha (1:3000, M20003H, Abmart), anti‐Myc (1:3000, M20002L, Abmart). Anti‐mouse secondary antibody (1:5000, A00160, GenScript), Anti‐rabbit secondary antibody (1:5000, A00098, GenScript).

### RNA Extraction and Quantitative Real‐Time PCR

Total RNA was isolated from cultured cells by using RNA isolater Total RNA Extraction Reagent (R401, Vazyme, China) and then converted to cDNA by using HiScript III 1st Strand cDNA Synthesis Kit (R312, Vazyme, China). The ATF4 and LAT1 mRNA levels were measured by quantitative real‐time PCR using ChamQ SYBR qPCR Master Mix (Q311, Vazyme, China) on the CFX Connect Real‐Time PCR Detection System (1 855 201, Bio‐Rad), with actin as an internal reference gene. The primers used for qRT‐PCR were:

ATF4: forward, 5′‐CTCTTGACCACGTTGGATGAC‐3′; reverse, 5′‐CAACTTCACTGCCTAGCTCTAAA‐3′LAT1: forward, 5′‐ATATCACGCTGCTCAACGGTG‐3′; reverse, 5′‐CTCCAGCATGTAGGCGTAGTC‐3′.

Actin: forward, 5′‐GTGACGTTGACATCCGTAAAGA‐3′; reverse, 5′‐GCCGGACTCATCGTACTCC‐3′.

### Acute DRG Neuron Dissociation

To perform patch clamp recordings in acutely isolated DRG neurons, the first prepared dissociated mice L3‐5 DRG neurons as per published methods.^[^ ^45^ ^]^ Isoflurane (4%) was used to euthanize the mice, and the lumbar section of the vertebral column was quickly dissected and immersed in chilled Hank's balanced salt solution without Mg^2+^ or Ca^2+^ (HBSS, Gibco). Then, the L3‐L5 DRG was isolated and dissociated with collagenase type I (1 mg mL^−1^) and dispase Il (5 mg mL^−1^) in HBSS for 30 min. Cells were re‐suspended in complete Neurobasal‐A Medium and added to the dishes with pre‐coated 8 mm coverslips (0.1 mg mL^−1^ poly‐D‐lysine, 20 µL per coverslip) and then placed into the incubator (37 °C, 5% CO_2_). All neurons were recorded 24 h after BCAA deficiency treatment.

### Whole‐Cell Patch Clamp Recording

Whole‐cell patch clamp recording was performed using borosilicate capillary glass pipettes (3‐5 MΩ by P‐97, Sutter Instruments) as per published methods.^[^ ^45^ ^]^ Cell cultures were plated into a thermostatic chamber (Warner, Instruments) mounted on an inverted microscope (Nikon, Japan) to maintain the temperature at 35 °C. The extracellular solution for Kv currents recording consisted of 145 mm NaCl, 3 mm KCI, 2 mm MgCI_2_, 2 mm CaCl_2_, 10 mm HEPES, and 10 mm glucose (pH 7.4 with NaOH, 315 mOsm with sucrose). The intracellular pipette solution for Kv currents and action potential recording contained 135 mm KCI, 2 mm MgCI_2_, 2 mm Na_2_‐ATP, 10 mm glucose, 10 mm HEPES, and 10 mm EGTA (pH 7.2 with KOH, 300 mOsm with sucrose). Tityustoxin‐Kα (5 mm) was used to block the Kv1.2 current. When recording the Kv current, 2 µM TTX, 0.1 mm CdCl_2_, and 5 mm 4‐AP were added to the extracellular solution to block Na^+^, Ca^2+^, and transient K^+^ currents, respectively. The total whole‐cell Kv current was stimulated by a 200 ms, −100 mV pre‐pulse, followed by a series of depolarizing voltages from −80 to +100 mV (200 ms) in 10‐mV increments at 1 s intervals. When recording action potentials by current‐clamp recording, the bridge was 100% balanced, and no holding current was injected into the neurons. A series of current pulses (0‐750 pA in steps of 25 pA, 200 ms) were injected to generate action potentials. Signals were low‐pass filtered at 2.9 kHz (EPC 10‐USB, HEKA, Germany) and digitized at 20 kHz using the Patchmaster software (HEKA). Online P/4 subtraction was used to remove the uncompensated membrane capacitance and leakage currents. The investigator was blinded to the treatment groups during the recording.

The Kv current density was calculated by dividing the whole‐cell current by the slow capacitance. The resting membrane potential (RMP) was calculated as the mean potential during the 10 ms before the first stimulus pulse in the initial trial. The action potential (AP) rheobase was defined as the minimum current required to evoke the first AP. The decay time was defined as the time required for the membrane potential to decrease from its peak value to 10% of its peak value.

### Quantification and Statistical Analysis

One‐way analysis of variance (ANOVA) was used to analyze the differences among three or more groups, followed by Bonferroni's multiple comparison test to obtain adjusted *p* values. Two‐tailed Student's *t*‐tests were performed for the two‐group analyses. All results were expressed as the mean ± SEM. *p* < 0.05 was considered statistically significant. Statistical analyses were performed using Prism software (version 10.0; GraphPad Software, Inc.) and Excel (Microsoft Corp.).

## Conflict of Interest

The authors declare no conflict of interest.

## Author Contributions

Z.Y.Z., J.Y.W., and Z.X.L. contributed equally to this work. The author contributions were as follows: J.Y.Z. and F.Q.L. conceived the study; J.Y.Z., F.Q.L., J.C., and Z.Q.P. designed and supervised the experiments; Z.Y.Z., J.Y.W., Z.X.L., and Y.N.Z. performed the experiments; Z.Y.Z., Z.X.L., X.S., K.C., Y.S., and A.F.C. analyzed the data; Z.Y.Z., H.L.Z., L.N.H., L.J.W., and X.W.D. generated the animal models; J.Y.W. and F.Q.L. collected the clinic samples. Z.Y.Z. and J.Y.Z. wrote the manuscript. All authors read and discussed the manuscript.

## Supporting information

Supporting Information

## Data Availability

The data that support the findings of this study are available from the corresponding author upon reasonable request.
