# Supplementary material for: Branched‐Chain Amino Acids Deficiency Promotes Diabetic Neuropathic Pain Through Upregulating LAT1 and Inhibiting Kv1.2 Channel
Source: Adv Sci (Weinh). 2024 Jul 1;11(33):2402086. doi: 10.1002/advs.202402086 (PMC11434239; doi:10.1002/advs.202402086)
Supplement: Supplementary file 1 — Supporting Information [file ADVS-11-2402086-s001.pdf]

ADVANCED SCIENCE
Open Access

Supporting Information

for *Adv. Sci.*, DOI 10.1002/advs.202402086

Branched-Chain Amino Acids Deficiency Promotes Diabetic Neuropathic Pain Through Upregulating LAT1 and Inhibiting Kv1.2 Channel

Ze-Yu Zhou, Ji-Ying Wang, Zhi-Xiao Li, Hong-Li Zheng, Ya-Nan Zhou, Li-Na Huang, Li-Juan Wang, Xiao-Wei Ding, Xin Sun, Ke Cai, Rui Zhao, Yan Shi, Alex F. Chen, Zhi-Qiang Pan, Jing Cao*, Fu-Qing Lin* and Jian-Yuan Zhao**

Figure S1

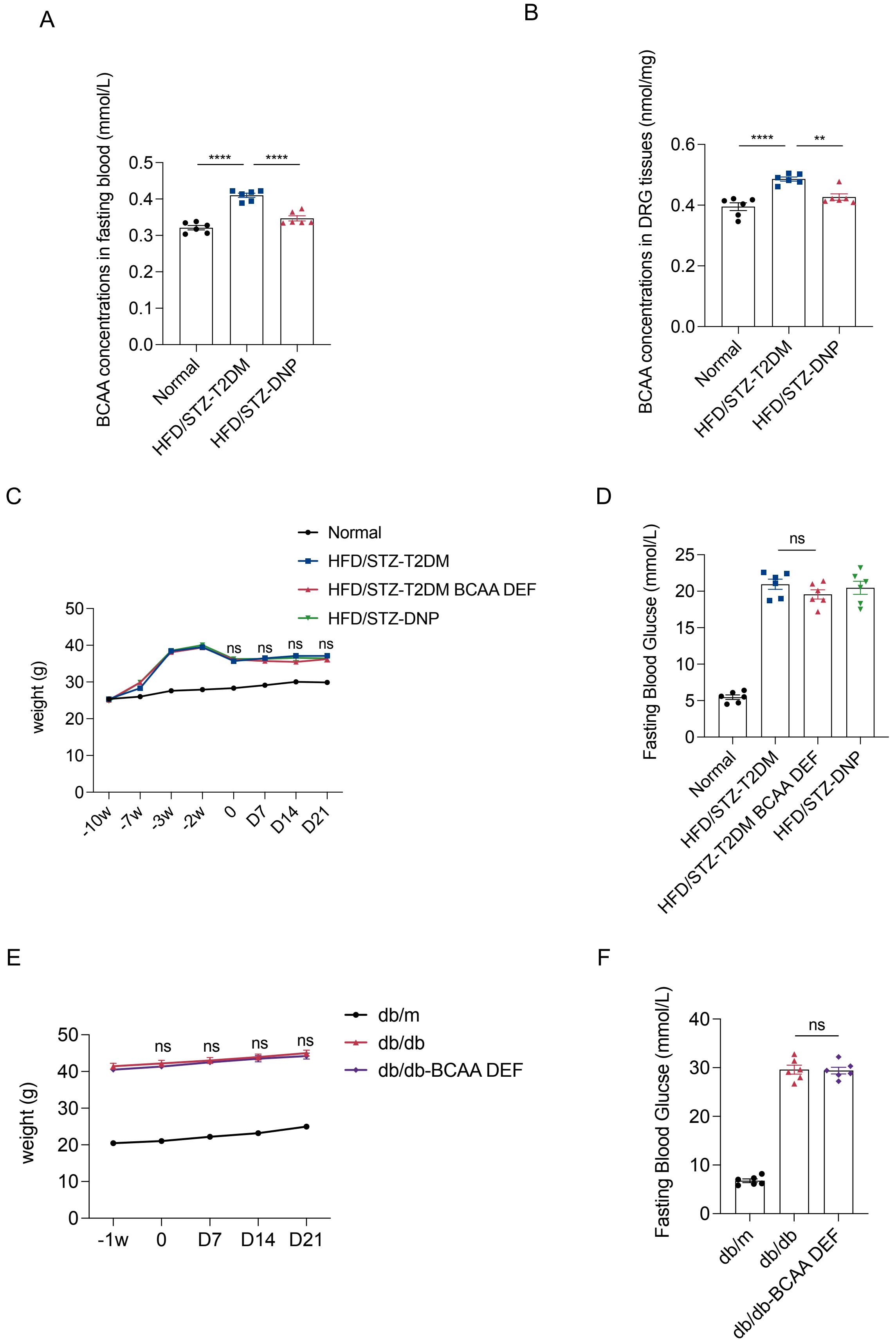
A
BCAA concentrations in fasting blood (mmol/L)
0.5
0.4
0.3
0.2
0.1
0.0
****
****
Normal
HFD/STZ-T2DM
HFD/STZ-DNP
B
BCAA concentrations in DRG tissues (nmol/mg)
0.6
0.4
0.2
0.0
****
**
Normal
HFD/STZ-T2DM
HFD/STZ-DNP
C
Normal
HFD/STZ-T2DM
HFD/STZ-T2DM BCAA DEF
HFD/STZ-DNP
weight (g)
50
40
30
20
10
0
ns
ns
ns
ns
-10w
-7w
-3w
-2w
0
D7
D14
D21
D
Fasting Blood Glucse (mmol/L)
25
20
15
10
5
0
ns
Normal
HFD/STZ-T2DM
HFD/STZ-T2DM BCAA DEF
HFD/STZ-DNP
E
db/m
db/db
db/db-BCAA DEF
weight (g)
50
40
30
20
10
0
ns
ns
ns
ns
-1w
0
D7
D14
D21
F
Fasting Blood Glucse (mmol/L)
40
30
20
10
0
ns
db/m
db/db
db/db-BCAA DEF

Figure S1. Information from HFD/STZ mice and db/db mice. Related to Figure 1.

A-B, BCAA concentrations in fasting blood (**A**) and DRG tissues (**B**) in the indicated groups (n=6 mice per group). **C-D**, Weight (**C**) and fasting blood glucose (**D**) of HFD/STZ mice in the indicated groups (n=6 mice per group). **E-F**, Weight (**E**) and fasting blood glucose (**F**) of db/db mice in the indicated groups (n=6 mice per group). Data shown are expressed as mean ± SEM. One-way analysis of variance (ANOVA) was used to analyze the differences among three or more groups, followed by Bonferroni's multiple comparison test to obtain adjusted *P* values. Differences between the HFD/STZ-T2DM and HFD/STZ-T2DM BCAA DEF groups, and between the db/db and db/db-BCAA DEF groups are shown. Significance is indicated as $^{ns}P>0.05$, $*P<0.05$, $**P<0.01$, $***P<0.001$, and $****P<0.0001$.

Figure S2

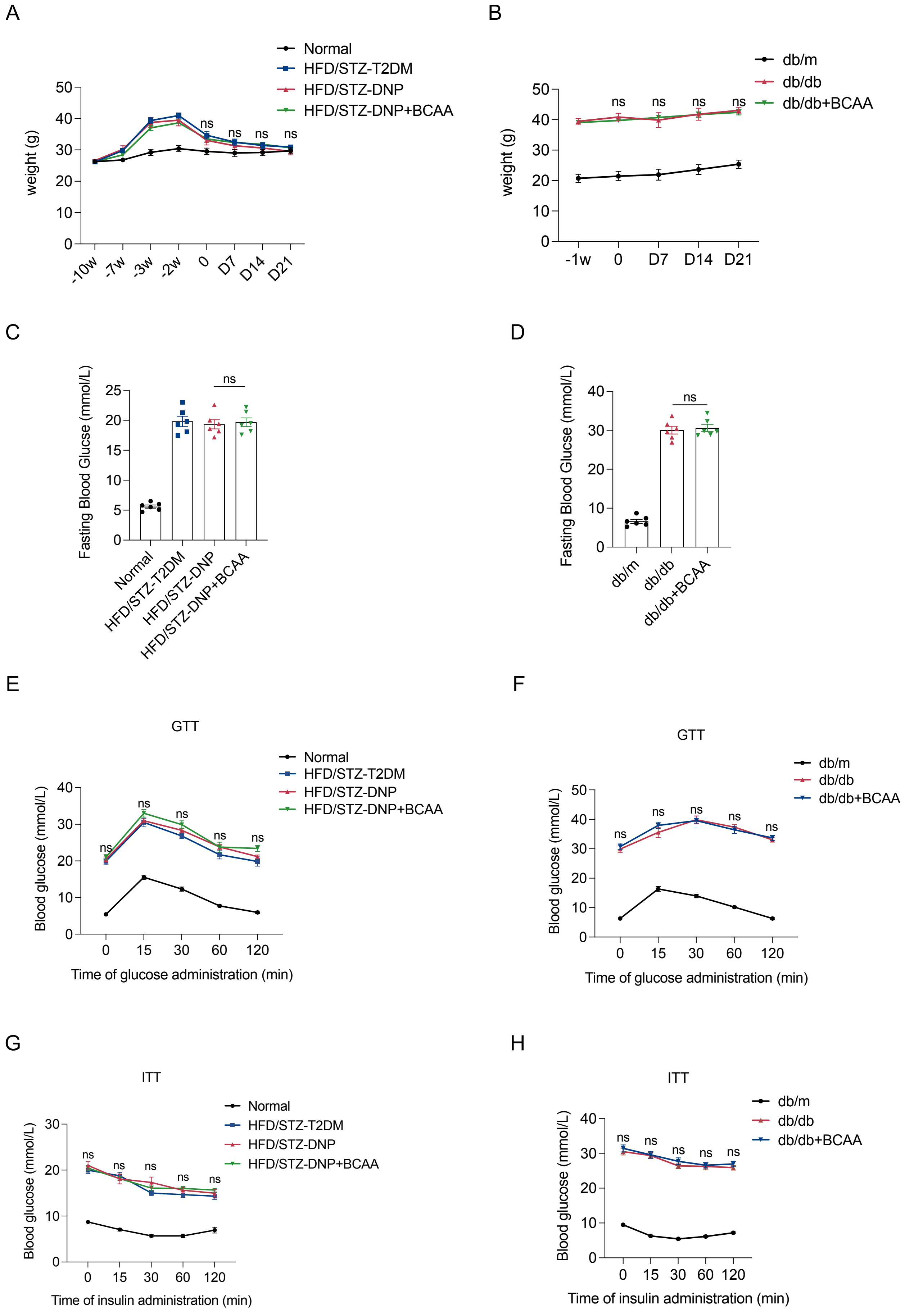
A
Normal
HFD/STZ-T2DM
HFD/STZ-DNP
HFD/STZ-DNP+BCAA
weight (g)
-10w
-7w
-3w
-2w
0
D7
D14
D21
ns
B
db/m
db/db
db/db+BCAA
weight (g)
-1w
0
D7
D14
D21
ns
C
Fasting Blood Gluose (mmol/L)
Normal
HFD/STZ-T2DM
HFD/STZ-DNP
HFD/STZ-DNP+BCAA
ns
D
Fasting Blood Glucose (mmol/L)
db/m
db/db
db/db+BCAA
ns
E
GTT
Normal
HFD/STZ-T2DM
HFD/STZ-DNP
HFD/STZ-DNP+BCAA
Blood glucose (mmol/L)
Time of glucose administration (min)
ns
F
GTT
db/m
db/db
db/db+BCAA
Blood glucose (mmol/L)
Time of glucose administration (min)
ns
G
ITT
Normal
HFD/STZ-T2DM
HFD/STZ-DNP
HFD/STZ-DNP+BCAA
Blood glucose (mmol/L)
Time of insulin administration (min)
ns
H
ITT
db/m
db/db
db/db+BCAA
Blood glucose (mmol/L)
Time of insulin administration (min)
ns

Figure S2. Information from HFD/STZ mice and db/db mice. Related to Figure 2.

A-B, Weight of HFD/STZ mice (**A**) and db/db mice (**B**) in the indicated groups (n=6 mice per group). **C-D**, Fasting blood glucose levels of HFD/STZ mice (**C**) and db/db mice (**D**) in the indicated groups (n=6 mice per group). **E-F**, Glucose tolerance was measured in fasted HFD/STZ mice (**E**) and db/db (**F**) mice after BCAA supplementation (n=6 mice per group). **G-H**, Insulin tolerance was measured in fasted HFD/STZ mice (**G**) and db/db mice (**H**) after BCAA supplementation (n=6 mice per group). Data shown are expressed as mean ± SEM. One-way analysis of variance (ANOVA) was used to analyze the differences among three or more groups, followed by Bonferroni's multiple comparison test to obtain adjusted *P* values. Differences between the HFD/STZ-DNP and HFD/STZ-DNP+BCAA groups, and between the db/db and db/db+BCAA groups are shown. Significance is indicated as $^{ns}P>0.05$, $^{*}P<0.05$, $^{**}P<0.01$, $^{***}P<0.001$, and $^{****}P<0.0001$.

Figure S3

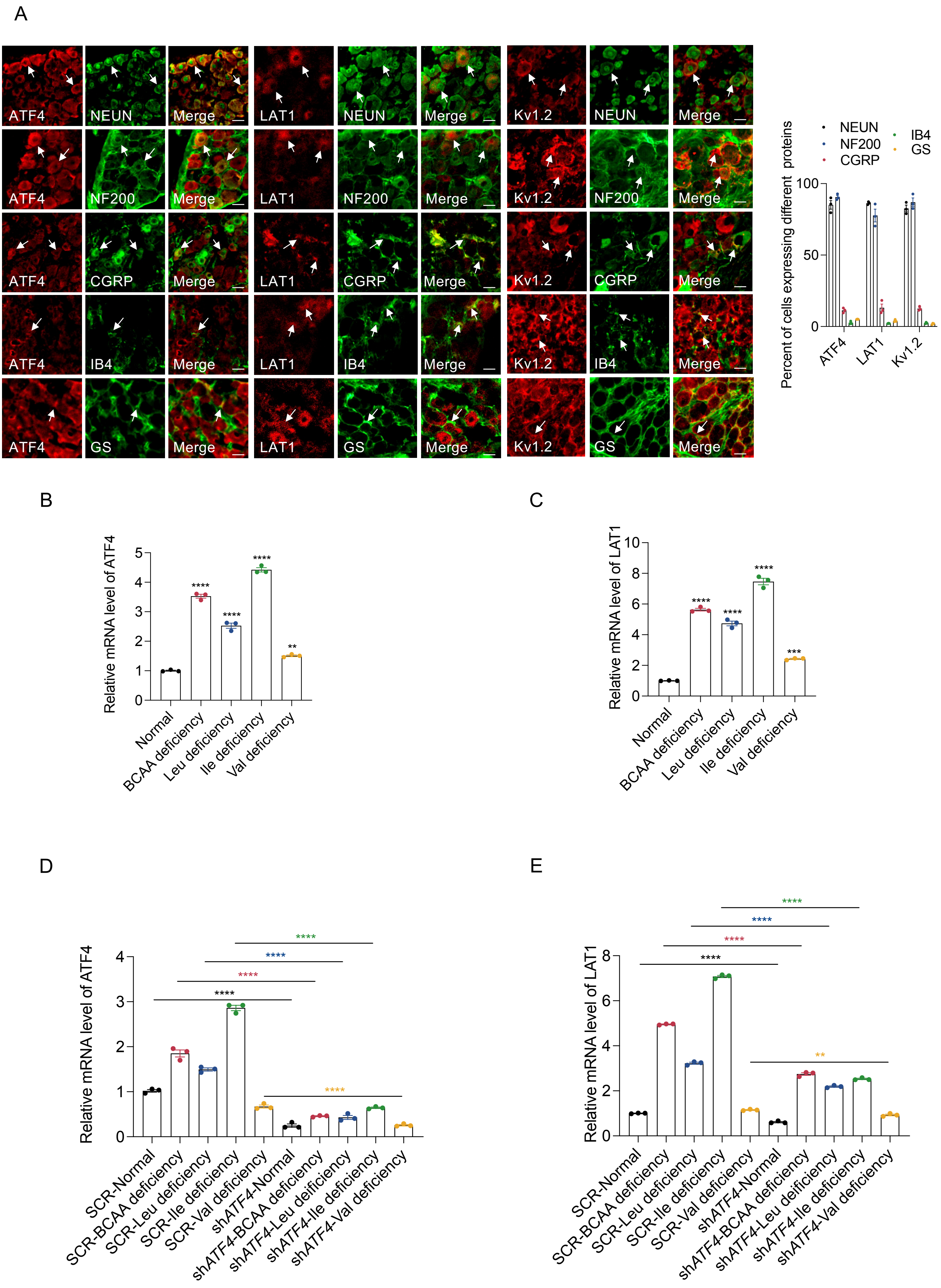
A
ATF4
NEUN
Merge
LAT1
Kv1.2
NF200
CGRP
IB4
GS
Percent of cells expressing different proteins
B
Relative mRNA level of ATF4
Normal
BCAA deficiency
Leu deficiency
Ile deficiency
Val deficiency
C
Relative mRNA level of LAT1
D
SCR-Normal
SCR-BCAA deficiency
SCR-Leu deficiency
SCR-Ile deficiency
SCR-Val deficiency
shATF4-Normal
shATF4-BCAA deficiency
shATF4-Leu deficiency
shATF4-Ile deficiency
shATF4-Val deficiency
E

Figure S3. BCAA deficiency compensates for LAT1 upregulation through ATF4. Related to Figure 4.

A, Representative fluorescence images of immunostaining for ATF4, LAT1, Kv1.2 and NEUN or NF200, or CGRP, or IB4 or GS in DRG neurons and glial cells. Quantification of immunostaining for ATF4, LAT1 and Kv1.2 expression levels are shown on the right. Scale bar: 200 μm. (n=3 per group). **B-C**, Relative mRNA levels of ATF4 (**B**) and LAT1 (**C**) in Neuro-2a cells treated with BCAA deficiency (n=3 per group). **D-E**, Relative mRNA levels of ATF4 (**D**) and LAT1 (**E**) in SCR and sh*ATF4* Neuro-2a cells treated with BCAA deficiency (n=3 per group). Data are expressed as mean ± SEM. One-way analysis of variance (ANOVA) was used to analyze the differences among three or more groups, followed by Bonferroni's multiple comparison test to obtain adjusted *P* values. Significance is indicated as $^{ns}P>0.05$, $^{*}P<0.05$, $^{**}P<0.01$, $^{***}P<0.001$, and $^{****}P<0.0001$.

Table S1. Clinical Characteristics of Participants

Characteristics	① Healthy People	② T2DM Patients	③ DNP Patients	② vs. ① *P* value	③ vs. ② *P* value
Number (n)	30	73	81	-	-
Age (years)	63.09 ± 0.71	61.05 ± 1.00	64.56 ± 1.22	0.873	0.055
Male, n (%)	26 (76.47%)	47 (61.04%)	52 (61.18%)	0.362	>0.999
Duration of Diabetes (years)	N/A	13.02 ± 0.83	14.61 ± 0.90	-	0.644
BMI (kg/m^2)	24.34 ± 0.33	25.69 ± 0.41	24.70 ± 0.42	0.187	0.225
FBG (mmol/L)	5.26 ± 0.09	7.64 ± 0.31	8.48 ± 0.31	3.24E-05	0.116
HbA1c (%)	N/A	9.03 ± 0.27	9.15 ± 0.24	-	0.735
SBP (mmHg)	131.74 ± 2.87	139.25 ± 2.19	134.94 ± 1.74	0.111	0.408
DBP (mmHg)	76.71 ± 2.03	78.35 ± 1.50	74.94 ± 0.90	>0.999	0.161
BNP (pg/mL)	N/A	35.51 ± 6.52	29.79 ± 3.80	-	0.435
ALB (g/L)	42.65 ± 0.40	40.49 ± 0.47	39.05 ± 0.50	0.031	0.077
TBil (μmol/L)	15.83 ± 1.22	12.51 ± 0.68	13.30 ± 1.46	0.309	>0.999
TBA (μmol/L)	N/A	5.77 ± 1.02	5.24 ± 0.97	-	0.709
ALT (U/L)	18.65 ± 1.21	26.93 ± 6.50	18.85 ± 1.29	0.794	0.473
AST (U/L)	19.28 ± 0.70	19.18 ± 1.53	16.94 ± 0.69	>0.999	0.406
ALP (U/L)	N/A	72.75 ± 2.81	68.60 ± 2.80	-	0.299
BUN (mmol/L)	N/A	6.64 ± 1.32	6.57 ± 0.35	-	0.956
Scr (μmol/L)	71.12 ± 1.20	65.93 ± 1.55	77.29 ± 4.75	>0.999	0.056
GFR (mL/min/1.73m^2)	96.44 ± 0.88	97.75 ± 1.83	92.30 ± 2.12	>0.999	0.106
UA (mmol/L)	N/A	315.16 ± 9.67	326.42 ± 9.51	-	0.408
Cys-C (mg/L)	N/A	0.89 ± 0.04	1.07 ± 0.10	-	0.082
TG (mmol/L)	1.44 ± 0.11	1.72 ± 0.14	1.55 ± 0.08	0.450	0.822
HDL (mmol/L)	1.21 ± 0.04	1.15 ± 0.04	1.15 ± 0.03	>0.999	>0.999
LDL (mmol/L)	3.16 ± 0.15	2.77 ± 0.10	2.53 ± 0.10	0.107	0.272
UMA (mg/24h)	N/A	32.34 ± 6.25	72.60 ± 22.55	-	0.114
1,25-$(OH)_2D_3$(ng/mL)	N/A	19.54 ± 6.96	19.25 ± 0.76	-	0.803
PTH (pg/mL)	N/A	28.21 ± 1.71	30.96 ± 1.89	-	0.284
FT3 (pmol/L)	N/A	4.98 ± 0.07	4.80 ± 0.07	-	0.062
FT4 (pmol/L)	N/A	15.87 ± 0.29	15.91 ± 0.32	-	0.915
TT3 (pmol/L)	N/A	1.50 ± 0.03	1.41 ± 0.03	-	0.059
TT4 (pmol/L)	N/A	100.27 ± 2.18	97.41 ± 2.35	-	0.379
TSH (mIU/L)	N/A	1.76 ± 0.14	2.07 ± 0.19	-	0.206
FCP (ng/mL)	N/A	1.98 ± 0.11	1.92 ± 0.14	-	0.756
NSE (ng/mL)	N/A	14.40 ± 0.51	14.16 ± 0.43	-	0.721

Abbreviations Notes: body mass index (BMI), fasting blood glucose (FBG), glycated hemoglobin A1c (HbA1c), systolic blood pressure (SBP), diastolic blood pressure (DBP), brain natriuretic peptide (BNP), albumin (ALB), total bilirubin (TBil), total bile acid (TBA), alanine aminotransferase (ALT), aspartate aminotransferase (AST), alkaline phosphatase (ALP), blood urea nitrogen (BUN), serum creatinine (Scr), glomerular filtration rate (GFR), uric acid (UA), cystatin C (Cys-C), triglyceride (TG), high- density lipoprotein (HDL), low-density lipoprotein (LDL), urinary microalbumin (UMA), 1,25-hydroxy vitamin D (1,25-$(OH)_2D_3$), parathyroid hormone (PTH), free triiodothyronine (FT3), free thyroxine (FT4), triiodothyronine (TT3), thyroxine (TT4), thyroid stimulating hormone (TSH), fasting C-peptide (FCP), and neuron- specific enolase (NSE).

Values are expressed as mean ± SEM. One-way analysis of variance (ANOVA) was used to analyze the differences among three groups, followed by Bonferroni's multiple comparison test to obtain adjusted *P* values. The comparisons between the two groups were performed using the nonparametric two-tailed Student's *t*-test.

Table S2. Medications Use of Participants

Medications	T2DM Patients	DNP Patients	*P* value
Metformin, n (%)	56 (69.14%)	66 (75.86%)	0.190
Insulin, n (%)	45 (55.56%)	55 (63.22%)	0.315
Acarbose, n (%)	24 (29.63%)	34 (39.08%)	0.200
Dapagliflozin, n (%)	15 (18.52%)	13 (14.94%)	0.537
Glimepiride, n (%)	14 (17.28%)	21 (24.14%)	0.277
Repaglinide, n (%)	8 (9.88%)	9 (10.34%)	0.921
Sitagliptin, n (%)	15 (18.52%)	13 (14.94%)	0.537
Pioglitazone, n (%)	16 (19.75%)	18 (20.69%)	0.881
Amlodipine, n (%)	21 (25.93%)	16 (18.39%)	0.242
Benazepril, n (%)	1 (1.23%)	3 (3.45%)	0.350
Valsartan, n (%)	17 (20.99%)	27 (31.03%)	0.141

The comparisons between the two groups were performed using the nonparametric two-tailed Student's *t*-test.

Table S3. Amino Acids Rodent Diet and Same Halved or Double BCAA

Diet#	FB-A10021B Control		FB-A10022 Halved BCAA		FB-A10022 Double BCAA	
	gm%	*kcal%*	gm%	*kcal%*	gm%	*kcal%*
Protein	17.00	*17.56*	15.60	*16.12*	19.80	*20.45*
Carbohydrate	68.55	*70.82*	69.95	*72.26*	65.75	*67.92*
Fat	5.00	*11.62*	5.00	*11.62*	5.00	*11.62*
Total	90.55	*100.00*	90.55	*100.00*	90.55	*100.00*
Ingredient(gm)	gm	*kcal*	gm	*kcal*	gm	*kcal*
L-Arginine	10	*40*	10	*40*	10	*40*
L-Histidine-HCl-H_2O	6	*24*	6	*24*	6	*24*
L-Isoleucine	8	*32*	4	*16*	16	*64*
L-Leucine	12	*48*	6	*24*	24	*96*
L-Valine	8	*32*	4	*16*	16	*64*
L-Tyrosine	4	*16*	4	*16*	4	*16*
L-Lysine-HCl	14	*56*	14	*56*	14	*56*
L-Methionine	6	*24*	6	*24*	6	*24*
L-Phenylalanine	8	*32*	8	*32*	8	*32*
L-Threonine	8	*32*	8	*32*	8	*32*
L-Tryptophan	2	*8*	2	*8*	2	*8*
L-Alanine	10	*40*	10	*40*	10	*40*
L-Asparagine-H_2O	5	*20*	5	*20*	5	*20*
L-Aspartate	10	*40*	10	*40*	10	*40*
L-Cystine	4	*16*	4	*16*	4	*16*
L-Glutamic Acid	30	*120*	30	*120*	30	*120*
L-Glutamine	5	*20*	5	*20*	5	*20*
Glycine	10	*40*	10	*40*	10	*40*
L-Proline	5	*20*	5	*20*	5	*20*
L-Serine	5	*20*	5	*20*	5	*20*
Corn Starch	550.5	*2202*	564.5	*2258*	522.5	*2090*
Maltodextrin	125	*500*	125	*500*	125	*500*
Cellulose	50	*0*	50	*0*	50	*0*
Corn Oil	50	*450*	50	*450*	50	*450*
Mineral Mix	35	*0*	35	*0*	35	*0*
Sodium Bicarbonate	7.5	*0*	7.5	*0*	7.5	*0*
Vitamin Mix	10	*40*	10	*40*	10	*40*
Choline Bitrartrate	2	*0*	2	*0*	2	*0*

Yellow Dye	0	*0*	0	*0*	0.025	*0*
Blue Dye	0.05	*0*	0.025	*0*	0.025	*0*
Red Dye	0	*0*	0.025	*0*	0	*0*
Total	1000.05	*3872*	1000.05	*3872*	1000.05	*3872*
